# The Association of Intraoperative driving pressure with postoperative pulmonary complications in open versus closed abdominal surgery patients – a posthoc propensity score–weighted cohort analysis of the LAS VEGAS study

**DOI:** 10.1186/s12871-021-01268-y

**Published:** 2021-03-19

**Authors:** Guido Mazzinari, Ary Serpa Neto, Sabrine N. T. Hemmes, Goran Hedenstierna, Samir Jaber, Michael Hiesmayr, Markus W. Hollmann, Gary H. Mills, Marcos F. Vidal Melo, Rupert M. Pearse, Christian Putensen, Werner Schmid, Paolo Severgnini, Hermann Wrigge, Oscar Diaz Cambronero, Lorenzo Ball, Marcelo Gama de Abreu, Paolo Pelosi, Marcus J. Schultz, for the LAS VEGAS study–investigators, Wolfgang Kroell, Helfried Metzler, Gerd Struber, Thomas Wegscheider, Hans Gombotz, Michael Hiesmayr, Werner Schmid, Bernhard Urbanek, David Kahn, Mona Momeni, Audrey Pospiech, Fernande Lois, Patrice Forget, Irina Grosu, Jan Poelaert, Veerle van Mossevelde, Marie-Claire van Malderen, Dimitri Dylst, Jeroen van Melkebeek, Maud Beran, Stefan de Hert, Luc De Baerdemaeker, Bjorn Heyse, Jurgen Van Limmen, Piet Wyffels, Tom Jacobs, Nathalie Roels, Ann De Bruyne, Stijn van de Velde, Brigitte Leva, Sandrine Damster, Benoit Plichon, Marina Juros-Zovko, Dejana Djonoviċ-Omanoviċ, Selma Pernar, Josip Zunic, Petar Miskovic, Antonio Zilic, Slavica Kvolik, Dubravka Ivic, Darija Azenic-Venzera, Sonja Skiljic, Hrvoje Vinkovic, Ivana Oputric, Kazimir Juricic, Vedran Frkovic, Jasminka Kopic, Ivan Mirkovic, Nenad Karanovic, Mladen Carev, Natasa Dropulic, Jadranka Pavicic Saric, Gorjana Erceg, Matea Bogdanovic Dvorscak, Branka Mazul-Sunko, Anna Marija Pavicic, Tanja Goranovic, Branka Maldini, Tomislav Radocaj, Zeljka Gavranovic, Inga Mladic-Batinica, Mirna Sehovic, Petr Stourac, Hana Harazim, Olga Smekalova, Martina Kosinova, Tomas Kolacek, Kamil Hudacek, Michal Drab, Jan Brujevic, Katerina Vitkova, Katerina Jirmanova, Ivana Volfova, Paula Dzurnakova, Katarina Liskova, Radovan Dudas, Radek Filipsky, Samir el Kafrawy, Hisham Hosny Abdelwahab, Tarek Metwally, Ahmed Abdel-Razek, Ahmed Mostafa El-Shaarawy, Wael Fathy Hasan, Ahmed Gouda Ahmed, Hany Yassin, Mohamed Magdy, Mahdy Abdelhady, Mohamed Mahran, Eiko Herodes, Peeter Kivik, Juri Oganjan, Annika Aun, Alar Sormus, Kaili Sarapuu, Merilin Mall, Juri Karjagin, Emmanuel Futier, Antoine Petit, Adeline Gerard, Emmanuel Marret, Marc Solier, Samir Jaber, Albert Prades, Jens Krassler, Simone Merzky, Marcel Gama de Abreu, Christopher Uhlig, Thomas Kiss, Anette Bundy, Thomas Bluth, Andreas Gueldner, Peter Spieth, Martin Scharffenberg, Denny Tran Thiem, Thea Koch, Tanja Treschan, Maximilian Schaefer, Bea Bastin, Johann Geib, Martin Weiss, Peter Kienbaum, Benedikt Pannen, Andre Gottschalk, Mirja Konrad, Diana Westerheide, Ben Schwerdtfeger, Hermann Wrigge, Philipp Simon, Andreas Reske, Christian Nestler, Dimitrios Valsamidis, Konstantinos Stroumpoulis, Georgios Antholopoulos, Antonis Andreou, Dimitris Karapanos, Kassiani Theodoraki, Georgios Gkiokas, Marios-Konstantinos Tasoulis, Tatiana Sidiropoulou, Foteini Zafeiropoulou, Panagiota Florou, Aggeliki Pandazi, Georgia Tsaousi, Christos Nouris, Chryssa Pourzitaki, Dmitri Bystritski, Reuven Pizov, Arieh Eden, Caterina Valeria Pesce, Annamaria Campanile, Antonella Marrella, Salvatore Grasso, Michele De Michele, Francesco Bona, Gianmarco Giacoletto, Elena Sardo, Luigi Giancarlo, Vicari Sottosanti, Maurizio Solca, Carlo Alberto Volta, Savino Spadaro, Marco Verri, Riccardo Ragazzi, Roberto Zoppellari, Gilda Cinnella, Pasquale Raimondo, Daniela La Bella, Lucia Mirabella, Davide D’antini, Paolo Pelosi, Alexandre Molin, Iole Brunetti, Angelo Gratarola, Giulia Pellerano, Rosanna Sileo, Stefano Pezzatto, Luca Montagnani, Laura Pasin, Giovanni Landoni, Alberto Zangrillo, Luigi Beretta, Ambra Licia Di Parma, Valentina Tarzia, Roberto Dossi, Marta Eugenia Sassone, Daniele Sances, Stefano Tredici, Gianluca Spano, Gianluca Castellani, Luigi Delunas, Sopio Peradze, Marco Venturino, Ines Arpino, Sara Sher, Concezione Tommasino, Francesca Rapido, Paola Morelli, Maria Vargas, Giuseppe Servillo, Andrea Cortegiani, Santi Maurizio Raineri, Francesca Montalto, Vincenzo Russotto, Antonino Giarratano, Marco Baciarello, Michela Generali, Giorgia Cerati, Yigal Leykin, Filippo Bressan, Vittoria Bartolini, Lucia Zamidei, Luca Brazzi, Corrado Liperi, Gabriele Sales, Laura Pistidda, Paolo Severgnini, Elisa Brugnoni, Giuseppe Musella, Alessandro Bacuzzi, Dalip Muhardri, Agreta Gecaj-Gashi, Fatos Sada, Adem Bytyqi, Aurika Karbonskiene, Ruta Aukstakalniene, Zivile Teberaite, Erika Salciute, Renatas Tikuisis, Povilas Miliauskas, Sipylaite Jurate, Egle Kontrimaviciute, Gabija Tomkute, John Xuereb, Maureen Bezzina, Francis Joseph Borg, Sabrine Hemmes, Marcus Schultz, Markus Hollmann, Irene Wiersma, Jan Binnekade, Lieuwe Bos, Christa Boer, Anne Duvekot, Bas in ‘t Veld, Alice Werger, Paul Dennesen, Charlotte Severijns, Jasper De Jong, Jens Hering, Rienk van Beek, Stefan Ivars, Ib Jammer, Alena Breidablik, Katharina Skirstad Hodt, Frode Fjellanger, Manuel Vico Avalos, Jannicke Mellin-Olsen, Elisabeth Andersson, Amir Shafi-Kabiri, Ruby Molina, Stanley Wutai, Erick Morais, Glória Tareco, Daniel Ferreira, Joana Amaral, Maria de Lurdes Goncalves Castro, Susana Cadilha, Sofia Appleton, Suzana Parente, Mariana Correia, Diogo Martins, Angela Monteirosa, Ana Ricardo, Sara Rodrigues, Lucian Horhota, Ioana Marina Grintescu, Liliana Mirea, Ioana Cristina Grintescu, Dan Corneci, Silvius Negoita, Madalina Dutu, Ioana Popescu Garotescu, Daniela Filipescu, Alexandru Bogdan Prodan, Gabriela Droc, Ruxandra Fota, Mihai Popescu, Dana Tomescu, Ana Maria Petcu, Marian Irinel Tudoroiu, Alida Moise, Catalin-Traian Guran, Iorel Gherghina, Dan Costea, Iulia Cindea, Sanda-Maria Copotoiu, Ruxandra Copotoiu, Victoria Barsan, Zsolt Tolcser, Magda Riciu, Septimiu Gheorghe Moldovan, Mihaly Veres, Alexey Gritsan, Tatyana Kapkan, Galina Gritsan, Oleg Korolkov, Alexander Kulikov, Andrey Lubnin, Alexey Ovezov, Pavel Prokoshev, Alexander Lugovoy, Natalia Anipchenko, Andrey Babayants, Irina Komissarova, Karginova Zalina, Valery Likhvantsev, Sergei Fedorov, Aleksandra Lazukic, Jasmina Pejakovic, Dunja Mihajlovic, Zuzana Kusnierikova, Maria Zelinkova, Katarina Bruncakova, Lenka Polakovicova, Villiam Sobona, Barbka Novak-Supe, Ana Pekle-Golez, Miroljub Jovanov, Branka Strazisar, Jasmina Markovic-Bozic, Vesna Novak-Jankovic, Minca Voje, Andriy Grynyuk, Ivan Kostadinov, Alenka Spindler-Vesel, Victoria Moral, Mari Carmen Unzueta, Carlos Puigbo, Josep Fava, Jaume Canet, Enrique Moret, Mónica Rodriguez Nunez, Mar Sendra, Andrea Brunelli, Frederic Rodenas, Pablo Monedero, Francisco Hidalgo Martinez, Maria Jose Yepes Temino, Antonio Martínez Simon, Ana de Abajo Larriba, Alberto Lisi, Gisela Perez, Raquel Martinez, Manuel Granell, Jose Tatay Vivo, Cristina Saiz Ruiz, Jose Antonio de Andrés Ibañez, Ernesto Pastor, Marina Soro, Carlos Ferrando, Mario Defez, Cesar Aldecoa Alvares-Santullano, Rocio Perez, Jesus Rico, Monir Jawad, Yousif Saeed, Lars Gillberg, Zuleyha Kazak Bengisun, Baturay Kansu Kazbek, Nesil Coskunfirat, Neval Boztug, Suat Sanli, Murat Yilmaz, Necmiye Hadimioglu, Nuzhet Mert Senturk, Emre Camci, Semra Kucukgoncu, Zerrin Sungur, Nukhet Sivrikoz, Serpil Ustalar Ozgen, Fevzi Toraman, Onur Selvi, Ozgur Senturk, Mine Yildiz, Bahar Kuvaki, Ferim Gunenc, Semih Kucukguclu, Şule Ozbilgin, Jale Maral, Seyda Canli, Oguzhan Arun, Ali Saltali, Eyup Aydogan, Fatma Nur Akgun, Ceren Sanlikarip, Fatma Mine Karaman, Andriy Mazur, Sergiy Vorotyntsev, Guy Rousseau, Colin Barrett, Lucia Stancombe, Ben Shelley, Helen Scholes, James Limb, Amir Rafi, Lisa Wayman, Jill Deane, David Rogerson, John Williams, Susan Yates, Elaine Rogers, Mark Pulletz, Sarah Moreton, Stephanie Jones, Suresh Venkatesh, Maudrian Burton, Lucy Brown, Cait Goodall, Matthew Rucklidge, Debbie Fuller, Maria Nadolski, Sandeep Kusre, Michael Lundberg, Lynn Everett, Helen Nutt, Maka Zuleika, Peter Carvalho, Deborah Clements, Ben Creagh-Brown, Philip Watt, Parizade Raymode, Rupert Pearse, Otto Mohr, Ashok Raj, Thais Creary, Ahmed Chishti, Andrea Bell, Charley Higham, Alistair Cain, Sarah Gibb, Stephen Mowat, Danielle Franklin, Claire West, Gary Minto, Nicholas Boyd, Gary Mills, Emily Calton, Rachel Walker, Felicity Mackenzie, Branwen Ellison, Helen Roberts, Moses Chikungwa, Clare Jackson, Andrew Donovan, Jayne Foot, Elizabeth Homan, Jane Montgomery, David Portch, Pauline Mercer, Janet Palmer, Jonathan Paddle, Anna Fouracres, Amanda Datson, Alyson Andrew, Leanne Welch, Alastair Rose, Sandeep Varma, Karen Simeson, Mrutyunjaya Rambhatla, Jaysimha Susarla, Sudhakar Marri, Krishnan Kodaganallur, Ashok Das, Shivarajan Algarsamy, Julie Colley, Simon Davies, Margaret Szewczyk, Thomas Smith, Ana Fernandez-Bustamante, Elizabeth Luzier, Angela Almagro, Marcos Vidal Melo, Luiz Fernando, Demet Sulemanji, Juraj Sprung, Toby Weingarten, Daryl Kor, Federica Scavonetto, Yeo Tze, the PROtective VEntilation NETwork, the Clinical Trial Network of the European Society of Anaesthesiology

**Affiliations:** 1 Research Group in Perioperative Medicine, Hospital Universitario y Politécnico la Fe, Avinguda de Fernando Abril Martorell 106, 46026 Valencia, Spain; 2 Department of Anesthesiology, Hospital Universitario y Politécnico la Fe, Valencia, Spain; 3 Department of Critical Care Medicine, Hospital Israelita Albert Einstein, São Paulo, Brazil; 4 Cardio-Pulmonary Department, Pulmonary Division, Faculdade de Medicina, Instituto do Coração, Hospital das Clinicas HCFMUSP, Universidade de Sao Paulo, Sao Paulo, Brazil; 5 Department of Intensive Care & Laboratory of Experimental Intensive Care and Anesthesiology (L·E·I·C·A), Academic Medical Center, Amsterdam, The Netherlands; 6 Department of Medical Sciences, Clinical Physiology, Uppsala University, Uppsala, Sweden; 7 PhyMedExp, INSERM U1046, CNRS UMR 9214, University of Montpellier, Montpellier, France; 8 Division Cardiac, Thoracic, Vascular Anesthesia and Intensive Care, Medical University Vienna, Vienna, Austria; 9 Operating Services, Critical Care and Anesthesia, Sheffield Teaching Hospitals, Sheffield and University of Sheffield, Sheffield, UK; 10 Department of Anesthesia, Critical Care and Pain Medicine, Massachusetts General Hospital, Boston, USA; 11 Queen Mary University of London, London, UK; 12 Department of Anesthesiology and Intensive Care Medicine, University Hospital Bonn, Bonn, Germany; 13 Department of Biotechnology and Sciences of Life, ASST- Settelaghi Ospedale di Circolo e Fondazione Macchi, University of Insubria, Varese, Italy; 14 Department of Anesthesiology, Intensive Care and Emergency Medicine, Pain Therapy, Bergmannstrost Hospital, Halle, Germany; 15 Policlinico San Martino Hospital – IRCCS for Oncology and Neurosciences, Genoa, Italy; 16 Department of Surgical Sciences and Integrated Diagnostics, University of Genoa Italy, Genoa, Italy; 17 Department of Anesthesiology and Intensive Care Therapy, Pulmonary Engineering Group, Technische Universität Dresden, Dresden, Germany; 18 Mahidol–Oxford Tropical Medicine Research Unit (MORU), Mahidol University, Bangkok, Thailand; 19 Nuffield Department of Medicine, University of Oxford, Oxford, UK

**Keywords:** Pneumoperitoneum, Laparoscopy, Laparoscopic surgery, Perioperative ventilation, Protective ventilation, PEEP, Respiratory mechanics, Driving pressure

## Abstract

**Background:**

It is uncertain whether the association of the intraoperative driving pressure (ΔP) with postoperative pulmonary complications (PPCs) depends on the surgical approach during abdominal surgery. Our primary objective was to determine and compare the association of time–weighted average ΔP (ΔP_TW_) with PPCs. We also tested the association of ΔP_TW_ with intraoperative adverse events.

**Methods:**

Posthoc retrospective propensity score–weighted cohort analysis of patients undergoing open or closed abdominal surgery in the ‘Local ASsessment of Ventilatory management during General Anaesthesia for Surgery’ (LAS VEGAS) study, that included patients in 146 hospitals across 29 countries. The primary endpoint was a composite of PPCs. The secondary endpoint was a composite of intraoperative adverse events.

**Results:**

The analysis included 1128 and 906 patients undergoing open or closed abdominal surgery, respectively. The PPC rate was 5%. ΔP was lower in open abdominal surgery patients, but ΔP_TW_ was not different between groups. The association of ΔP_TW_ with PPCs was significant in both groups and had a higher risk ratio in closed compared to open abdominal surgery patients (1.11 [95%CI 1.10 to 1.20], *P* <  0.001 versus 1.05 [95%CI 1.05 to 1.05], *P* <  0.001; risk difference 0.05 [95%CI 0.04 to 0.06], *P* <  0.001). The association of ΔP_TW_ with intraoperative adverse events was also significant in both groups but had higher odds ratio in closed compared to open abdominal surgery patients (1.13 [95%CI 1.12– to 1.14], *P* <  0.001 versus 1.07 [95%CI 1.05 to 1.10], *P* <  0.001; risk difference 0.05 [95%CI 0.030.07], *P* <  0.001).

**Conclusions:**

ΔP is associated with PPC and intraoperative adverse events in abdominal surgery, both in open and closed abdominal surgery.

**Trial registration:**

LAS VEGAS was registered at clinicaltrials.gov (trial identifier NCT01601223).

**Supplementary Information:**

The online version contains supplementary material available at 10.1186/s12871-021-01268-y.

## Introduction

The incidence of postoperative pulmonary complications (PPCs) is high and depends on the used definitions and the studied population [1]. Their occurrence is associated with increased morbidity and mortality [2, 3]. PPCs can be prevented by reducing lung strain by using a low tidal volume (V_T_) [4], ,and by using sufficient positive end–expiratory pressure (PEEP) [5]. Since the driving pressure (ΔP), defined as the difference between plateau pressure and PEEP, is associated with the development of PPCs [5, 6], titrating V_T_ and PEEP to obtain the lowest ΔP could be an effective preventive strategy against PPCs.

The overall behaviour of the respiratory system depends on the properties of its components, i.e., the artificial and native airways, and the lung tissue, but also the chest wall consisting of the rib cage and diaphragm. Most of the force applied during invasive ventilation is needed to expand the chest wall, and only a lesser amount to inflate lung tissue [7]. When the chest wall elastance increases, e.g., during pneumoperitoneum, the ΔP increases, even when V_T_ is left unchanged [8]. This rise in ΔP is often interpreted as ‘innocent’, and therefore accepted during intraoperative pneumoperitoneum. However, the cephalad shift of the diaphragm could induce, or worsen atelectases during intraoperative ventilation, and the resulting increase in ΔP is related with a rise in lung applied force [9]. In other words, it should be questioned if a rise in ΔP during pneumoperitoneum with closed abdominal surgery can be accepted.

To determine and compare the independent associations of ΔP with PPCs in patients undergoing open abdominal surgery versus patients undergoing closed abdominal surgery, we reassessed the database of the ‘Local ASsessment of Ventilatory management during General Anaesthesia for Surgery’ (LAS VEGAS) study [10]. The LAS VEGAS study was a large observational study that included a large proportion of patients at an increased risk for PPCs. The primary hypothesis tested here was that the association of ΔP with PPCs is weaker in closed versus open abdominal surgery patients. The primary objective was to test the association of a time–weighted average driving pressure (ΔP_TW_) with PPCs. The secondary objective was to test the association of ΔP_TW_ with intraoperative adverse events.

## Methods

### Study design and setting

This is a posthoc analysis of the LAS VEGAS study [10], carried out following current guidelines and the recommendations of the statement for strengthening the reporting of observational studies in epidemiology (STROBE) (www.strobe-statemenent.org). The statistical analysis plan was predefined, updated, and finalised before data extraction, and is presented as Additional file 1. The LAS VEGAS study is a worldwide international multicentre prospective seven–day observational study describing intraoperative ventilation practice, complications during anaesthesia, PPCs in the first five postoperative days, hospital length of stay, and hospital mortality.

The ethical committee of the Academic Medical Center, Amsterdam, the Netherlands, approved the LAS VEGAS study protocol (W12_190#12.17.0227). Each participating centre obtained approval from their institutional review board if needed, and patients were included after obtaining written informed consent when dictated by national or regional legislation. The LAS VEGAS study was partially funded and endorsed by the European Society of Anaesthesiology and registered at clinicaltrials.gov (study identifier NCT01601223, first posted date: 17/05/2012).

### Inclusion and exclusion criteria

The LAS VEGAS study recruited consecutive patients undergoing general anaesthesia with mechanical ventilation during anaesthesia for surgery during a seven–days timeframe between 14 January and 4 March 2013. Exclusion criteria of the LAS VEGAS study were: (1) age < 18 years, (2) having received mechanical ventilation in the preceding month, (3) obstetric or ambulatory surgical interventions, and (4) cardiothoracic surgery cardiopulmonary bypass.

For the current analysis, inclusion was restricted to patients undergoing abdominal surgery. The following additional exclusion criteria were used: (1) insufficient data to calculate ΔP, i.e., on at least two timepoints sufficient data had to be available to calculate the driving pressure for a patient to be included; (2) to increase the homogeneity of the compared patient cohorts and avoid using erroneous data, patients who received intraoperative ventilation through an airway device other than an endotracheal tube as well as patients under an assisted or spontaneous ventilation mode were excluded; (3) patients in whom laparoscopy only assisted the surgery, i.e., surgeries that could not be classified as mere open or mere closed abdominal surgery, were also excluded from the current analysis.

### Data recording and calculations

Full details on data collection can be found in the original publication of the LAS VEAGS study [10], and in Additional file 2. In the LAS VEGAS study database, ventilatory parameters at every hour of surgery, from induction up to the last hour of surgery, were recorded. Data in the LAS VEGAS database was validated through two rounds of extensive data cleaning to check for invalid or incomplete data. Local investigators were queried on incorrect or missing data and had to correct those in the cleaning rounds.

The following calculations were performed. ΔP was calculated by subtracting PEEP from plateau pressure or inspiratory pressure at every hour in volume–controlled and pressure–controlled ventilated patients, respectively. ΔP_TW_, i.e., the pressure that is proportional to the amount of time spent at each driving pressure in relation to the total time, was calculated by summing the mean values between consecutive time points multiplied by the time between those points and then dividing by the entire time [11]. Similarly, time–weighted average peak pressure and PEEP were determined. Details on calculations are provided in the Additional file 2 Figure S1.

### Definitions

PPCs were defined as a collapsed composite of the following events: unexpected postoperative invasive or non–invasive ventilation, acute respiratory failure, acute respiratory distress syndrome, pneumonia, and pneumothorax. The occurrence of each type of complication was monitored until hospital discharge but restricted to the first five postoperative days.

Intraoperative adverse events were defined as an ordinal composite of the following events: any oxygen desaturation or lung recruitment manoeuvres performed to rescue from hypoxemia, any need for adjusting ventilator settings for reducing airway pressures or correction of expiratory flow limitation, any hypotension or need for vasoactive drugs, and any new cardiac arrhythmia.

A detailed list of definitions of the composites of PPCs and intraoperative adverse events is provided in Additional file 2 Table S1 and Table S2.

### Endpoints

The primary endpoint was the composite of PPCs. The secondary endpoint was the composite of intraoperative adverse events.

### Analysis plan

The analysis plan was prespecified before data access, and we used data of all available patients in the LAS VEGAS database without formal sample size calculation. Also, as the purpose of the analysis was exploring a physiological hypothesis, we did not specify any a priori effect size.

Continuous variables were reported as median and interquartile ranges; categorical variables expressed as n (%). Normality of distributions was assessed by inspecting quantile–quantile plots. If variables were normally distributed, the two–sample t–test was used; if not, the Wilcoxon rank sum test was used. We used the Chi–square test or Fisher’s exact test for categorical variables, or when appropriate, as relative risks. Statistical uncertainty was expressed by showing the 95%–confidence intervals (CI). Since the simultaneous occurrence of various intraoperative adverse events is frequent, we analysed them as an ordinal variable with a range spanning from zero to seven adverse events.

To control for confounding effects, we estimated the association of ΔP_TW_ with PPC with a weighted mixed–effect logistic regression, and the association of ΔP_TW_ with intraoperative adverse events with a weighted mixed ordinal regression. To fit the models, we introduced centres as a random intercept, and an inverse probability weighting factor computed from the covariate–balancing propensity score (CBPS) method to simultaneously optimise treatment assignment prediction, i.e., ΔP_TW_ as a continuous variable, and confounders influence [12]. The CBPS procedure sets mean independence between treatment, i.e., ΔP_TW_, and covariates to ensure covariate balancing and estimates the propensity score with the generalised method of moments method. For both outcomes, we fitted the model for each of the compared patient cohorts respectively, i.e., patients who underwent open surgery intervention and those who underwent closed surgical intervention. We used a Wald z-test to test the difference between odds ratios from models fitted on closed and open surgery cohort. Models’ goodness of fit was assessed by residual diagnosis based on scaled quantile residuals (R *DHARMa* package v. 0.2.4) and simulated residuals (R *sure* package v 0.2.0) for logistic and ordinal models, respectively.

To build the CBPS to relate exposure variable, i.e., ΔP_TW_, with potential confounders, we included by clinical judgment the Assess Respiratory Risk in Surgical Patients in Catalonia (ARISCAT) risk class [13, 14], and the average intraoperative V_T._ Then we performed feature selection with an augmented backward elimination selection method introducing 37 preoperative and intraoperative variables (Additional file 2:Statistics for a detailed list). The selection was based on a sequential process where initially all variables entered the model and finally those preoperative and intraoperative factors that yielded a change in the effect estimate > 0.1 and a significance criterion (alpha) <  0.1 were included. The algorithm stopped when all variables left in the model complied with both criteria [15]. We carried out a selection process of potential variables to avoid bias in the effect estimates using a comprehensive strategy to prevent the drawbacks of simple stepwise methods [16]. The model’s internal validation was assessed by bootstrap using 5 hundred generated samples and estimating the Area Under Curve (AUC) of the full and stepwise–selected variables models.

To further unravel the effect of the surgical approach on PPCs, we performed a sensitivity analysis fitting a mixed logistic regression with a random intercept for centre on a propensity score matched cohort. The propensity score was used to match patients with a similar covariable structure using the R *matchit* package carrying out the matching with the nearest neighbour method with a caliper of 0.1 with a ratio of patients in the open surgery arm of 2 to 1. Full details on the covariables introduced in the propensity score matching procedure can be found in the Additional file 2**:** Statistics. To assess the type of surgery as an effect modifier, we carried out another sensibility analysis fitting a weighted mixed logistic regression model on all data, i.e., both surgery cohorts, introducing the type of surgery as an independent variable and an interaction term between ΔP_TW_ and type of surgery.

Statistical significance was considered for two–tailed *P* <  0.05. No imputation routine of missing values and no correction for multiple comparisons was prespecified; thus, all the findings should be viewed as exploratory. All analyses were performed with R 3.5.2 (The R Foundation for Statistical Computing, www.r-project.org). Additional explanation on the used methods can be found in the Additional file 2: Statistics.

## Results

### Patients

Of a total of 3265 patients undergoing abdominal surgery in the LAS VEGAS study, 1231 had insufficient data for calculating the ΔP (37.7%).

**Fig. 1:**
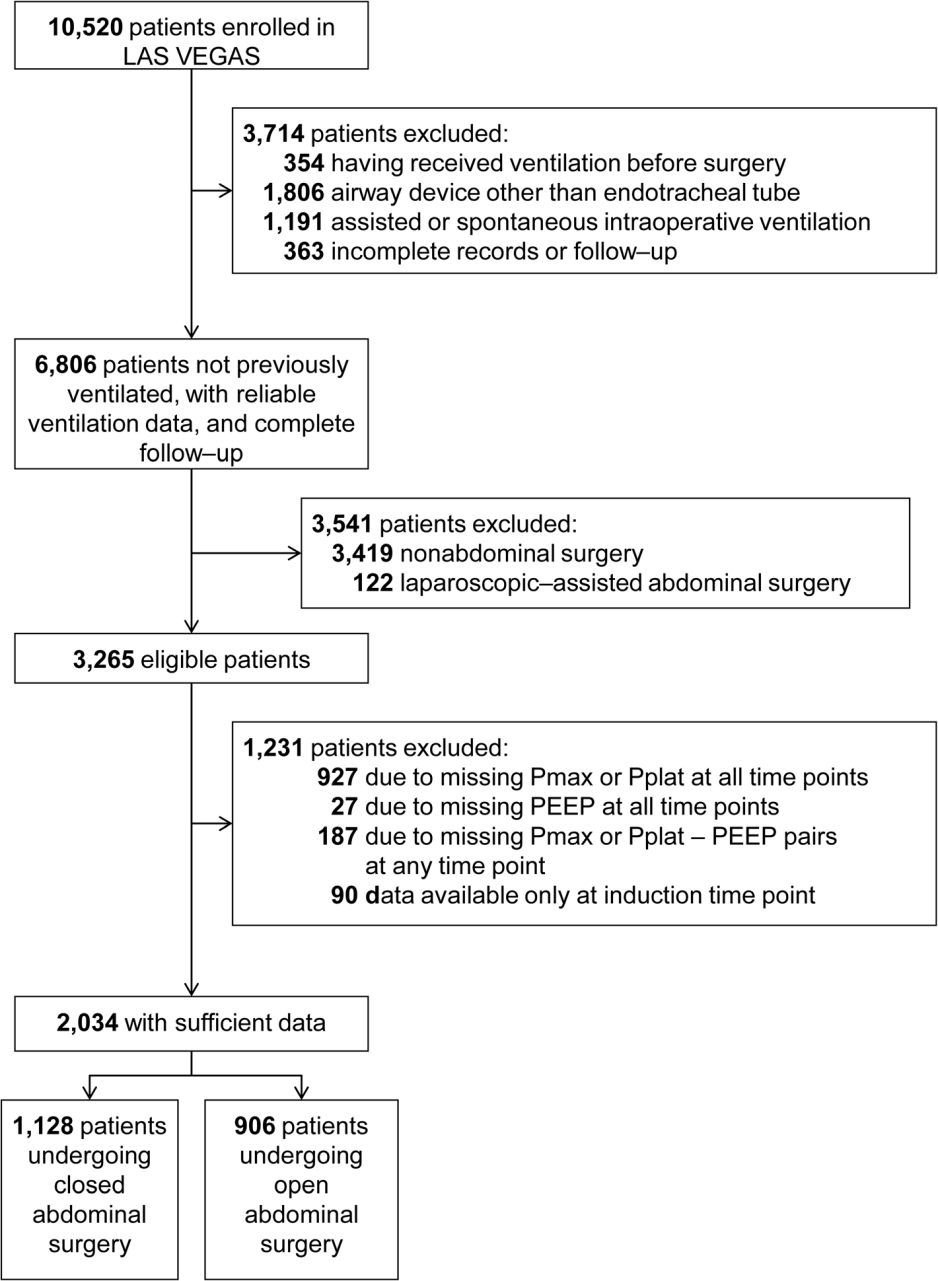
Patients’ inclusion flowchart

**Fig. 2:**
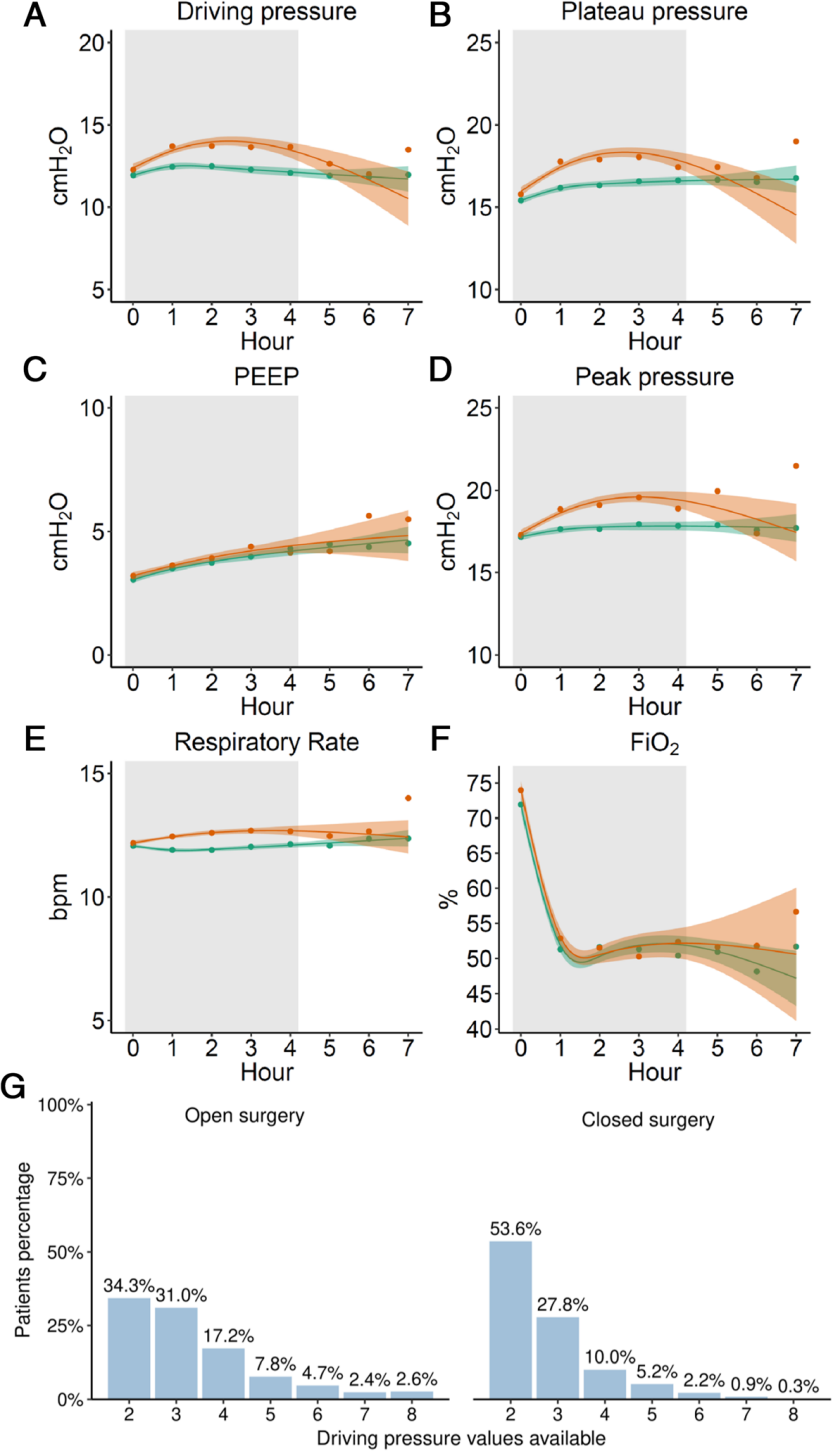
Mechanical ventilation settings over time. Green: open surgery, Orange: closed surgery. Hour 0 h represents the induction of general anaesthesia. Solid lines are means, and bandwidths is 95% bootstrapped confidence intervals. Gray boxes: More than 95% of data points represented

Out of the remaining 2034 patients, 1218 (60%) patients underwent an open abdominal intervention, and 906 (40%) patients, a closed abdominal surgical procedure (Fig. 1). ΔP could be calculated on two different timepoints in 34.4 and 53.7% of patients in the open and closed surgery group, respectively (Fig. 2 and Table S3). In 87% of patients, ΔP could be calculated on up to four timepoints.

Baseline demographic data, surgery–related and intraoperative ventilation characteristics are presented in Tables 1 and 2, and Fig. 2. Open abdominal surgery patients had higher ASA class and ARISCAT risk score, lower functional status, and fewer elective procedures, longer surgery times, less neuromuscular reversals, and received more intraoperative transfusions and fluids. Lower abdomen surgeries were the most frequently performed in the open abdominal surgery patients, while upper abdomen interventions were performed more often in closed abdominal surgery patients. ΔP_TW_ was not different between the open and closed surgery groups (Table 2).

**Table 1:** Patients demographics and surgery–related characteristics

	All patients (***N*** = 2.034)	Closed abdominal surgery (***N*** = 906)	Open abdominal surgery (***N*** = 1.128)	***P***–value	Absolute Difference
**Age, years**	54 [40 to 67]	49 [36 to 64]	58 [45 to 69]	< 0.001	9 [6 to 21]
**Gender, male (%)**	42% (846/2034)	34% (306/906)	48% (540/1128)	< 0.001	14% [9 to 18%]
**Ethnicity, % (n/N)**				0.194	
Caucasian	88% (1787/2.030)	87% (786/902)	89% (1001/1.128)		
Black	1% (20/2.030)	1% (6/902)	1% (14/1.128)		
Asian	3% (58/2.030)	4% (33/902)	2% (25/1.128)		
Other	8% (165/2.030)	8% (77/902)	8% (88/1.128)		
**BMI (Kg∙m**^**−2**^**)**	26.2 [23.3 to 30.0]	26.7 [23.6 to 31.3]	25.8 [22.9 to 29.3]	< 0.001	0.8 [0.04 to 1.6]
**Weight (kg)**	75.0 [65.0 to 87.0]	77.0 [68.0 to 93.0]	74.0 [64.0 to 85.0]	0.001	3 [8 to 13]
**PBW (kg)**	60.6 [55.1 to 69.0]	59.7 [54.2 to 67.8]	61.5 [56.0 to 69.7]	< 0.001	1.82 [1.8 to 2]
**ASA class, % (n/N)**				< 0.001	
1	24% (495/2.028)	31% (276/904)	20% (219/1.124)		
2	49% (989/2.028)	53% (477/904)	46% (512/1.124)		
3	24% (488/2.028)	16% (146/904)	30% (342/1.124)		
4	3% (53/2.028)	1% (5/904)	4% (48/1.124)		
5	0% (3/2.028)	0% (0/904)	0% (3/1.124)		
**ARISCAT score**	26 [18 to 38]	18 [15 to 31]	34 [18 to 41]	< 0.001	16 [16 to 16]
**ARISCAT class, % (n/N)**				< 0.001	
< 26	51% (985/1.945)	68% (607/888)	36% (378/1.057)		
26–44	38% (741/1.945)	26% (231/888)	48% (510/1.057)		
> 44	11% (219/1.945)	6% (50/888)	16% (169/1.057)		
**Preop. SpO**_**2**_**,%**	98 [96 to 99]	98 [96 to 99]	97 [96 to 99]	0.004	0 [0 to 3]
**Current smoker, %**	20% (413/2.034)	21% (79/906)	20% (222/1.128)	0.468	2% [3 to 7%]
**Chronic comorbidity, % (n/N)**					
Metastatic cancer	7% (138/2.034)	2% (22/906)	10% (116/1.128)	< 0.001	8% [5 to 9%]
Chronic kidney failure	4% (81/2.034)	1% (13/906)	6% (68/1.128)	< 0.001	5% [2 to 6%]
COPD	7% (138/2.034)	7.% (83/906)	6% (55/1.128)	0.290	1% [1 to 3%]
Heart failure	7% (143/2.034)	6% (53/906)	8% (90/1.128)	0.075	2% [1 to 4%]
OSAS	2% (42/2.034)	3% (27/906)	1% (15/1.128)	0.015	2% [1 to 3%]
Neuromuscular disease^a^	1% (17/2.034)	1% (6/906)	1% (11/1.128)	0.599	0.3% [0.3 to 1%]
Liver dysfunction	1% (29/2.034)	1% (5/906)	2% (24/1.128)	0.210	1% [1 to 2%]
**Functional Status, % (n/N)**				< 0.001	
Independent	92% (1872/2.034)	96% (867/906)	89% (1005/1.128)		
Partially dependent	7% (135/2.034)	4% (32/906)	9% (103/1.128)		
Totally dependent	1% (27/2.034)	1% (7/906)	2% (20/1.128)		
**Preop. resp. infection,% (n/N)**	5% (95/2.034)	4% (35/906)	5% (60/1.128)	0.150	2% [0.5 to 3%]
**Preop. Hb (g∙dl**^**−1**^**), % (n/N)**	13.4 [12.2 to 14.0]	13.5 12.6 to 14.5]	13.3 [11.9 to 14.5]	< 0.001	0.2 [0.3 to 1]
**Preop. anemia (Hb ≤ 10 g dl**^**−1**^**)**	9% (1738/1.846)	3% (21/798)	8% (87/1.048)	< 0.001	5% [3 to 7%]
**Preop. creatinine (g∙dl**^**− 1**^**)**	0.8 [0.7 to 1.0]	0.8 [0.7 to 1.0]	0.9 [0.7 to 1.1]	< 0.001	0.04 [0.01 to 0.1]
**Preop transfusion, % (n/N)**	1% (23/2.034)	0% (3/906)	2% (20/1.128)	0.004	1% [0.5 to 1%]
**Surgical procedure**^b^, **% (n/N)**					
Lower GI	26% (286/1.098)	14% (124/906)	31% (346/1.128)	< 0.001	17% [13 to 20%]
Upper GI, HBP	28% (303/1.098)	47% (429/906)	20% (222/1.128)	< 0.001	27% [23 to 31%]
Vascular surgery	2% (25/1.098)	0% (0/906)	3% (30/1.128)	< 0.001	2% [1 to 3%]
Aortic surgery	2% (19/1.098)	0% (0/906)	2% (20/1.128)	< 0.001	1% [1 to 2%]
Urological	19% (204/1.098)	9% (81/906)	14% (162/1.128)	< 0.001	5% [2 to 8%]
Gynaecological	18% (195/1.098)	26% (233/906)	17% (188/1.128)	< 0.001	9% [6 to 12%]
Endocrine surgery	1% (9/1.098)	1% (5/906)	1% (10/1.128)	0.443	0.3% [0.5 to 1%]
Transplant	2% (18/1.098)	0% (0/906)	2% (20/1.128)	< 0.001	2% [1 to 3%]
Neurosurgery	5% (52/1.098)	0% (1/906)	10% (109/1.128)	< 0.001	9% [8 to 11%]
Other procedure	3% (30/1.098)	5% (43/906)	19% (214/1.128)	< 0.001	14% [11 to 17%]
**Urgency of Surgery**^c^, **% (n/N)**				< 0.001	
Elective	84% (1705/2.034)	87% (792/906)	81% (913/1.128)		
Urgent	12% (235/2.034)	9% (85/906)	13% (150/1.128)		
Emergency	4% (94/2.034)	4% (29/906)	6% (65/1.128)		
**Duration of surgery**^**d**^**, min**	86 [55 to 149]	70 [50 to 110]	105 [65 to 172]	< 0.001	35 [21 to 43]
**Duration of anaesthesia**^**e**^**, min**	115 [80 to 190]	100 [71 to 147]	140 [91 to 205]	< 0.001	40 [20 to 60]
**Time of surgery**, **% (n/N)**				< 0.843	0.2 [0.2 to 1]
Daytime^f^	95% (1925/2034)	95% (859/906)	95%(1066/1128)		
Night–time	5% (109/2034)	5% (47/906)	5% (962/1128)		
**Antibiotic prophylaxis, % (n/N)**	80% (1.628/2.034)	73% (662/906)	84% (956/1.127)	0.005	11% [8 to 15%]
**Mean arterial pressure, mmHg**	82 [74 to 92]	84 [76 to 94]	80 [72 to 90]	< 0.001	4 [4 to 7]
**Heart rate, beats∙min**	72 [63 to 82]	73 [64 to 82]	72 [62 to 83]	0.276	1 [3 to 11]
**Intraop. procedures, % (n/N)**					
Epidural anesthesia	12% (237/2.034)	3% (25/906)	19% (212/1128)	< 0.001	16% [13 to 18%]
Opioid				< 0.001	
Short–acting	18% (367/2.015)	22% (193/900)	16% (174/1.115)		
Long–acting	70% (1410/2.015)	62% (561/900)	76% (849/1.115)		
Both	12% (238/2.015)	16% (146/900)	8% (92/1.115)		
Neuromuscular Blockade	97% (1965/2.028)	97% (876/903)	97% (1089/1.125)	0.887	0.2% [0.1 to 1%]
Neuromuscular Monitoring	23% (474/2.032)	25% (230/906)	22% (244/1.126)	0.055	3% [0 to 7%]
Neuromuscular Reversal	41% (827/2.024)	49% (437/901)	35% (390/1.123)	< 0.001	14% [9 to 18%]
TIVA	10% (211/2.027)	11% (102/902)	10% (109/1.125)	0.266	1% [1 to 4%]
Transfusion	6% (113/2.034)	1% (13/906)	9% (100/1.128)	< 0.001	7% [6 to 9%]
Total Fluids (mL∙ kg^−1^)	18 [12 to 30]	15 [13 to 30]	23 [14 to 26]	< 0.001	8 [6 to 10]
Crystalloids (mL∙ kg^−1^)	17 [12 to 26]	14 [11 to 21]	20 [13 to 31]	< 0.001	5 [4 to 7]
Colloids (mL∙ kg^−1^)	7 [3 to 9]	4 [0 to 7]	7 [6 to 12]	< 0.001	3 [2 to 6]

Data are presented as median [25th–75th percentile] or % (n/N). For binary and continuous variables risk difference and median difference with 95% confidence intervals in square brackets are reported respectively

Abbreviations: *BMI* Body mass index, *ASA* American Society of Anaesthesiologists, *ARISCAT* Assess Respiratory Risk in Surgical Patients in Catalonia risk index,^14,15^ *Hb* Haemoglobin, *GI* Gastrointestinal, *HBP* Hepatobiliopancreatic, *SpO*_*2*_ Peripheral oxygen saturation, *CI* Confidence interval, *COPD* Chronic Obstructive Pulmonary Disease, *OSAS* Obstructive sleep apnea sydnrome, *TIVA* Total Intravenous Anaesthesia

^a^Neuromuscular disease affecting the respiratory system

^b^The same patient may have more than one surgical indication

^c^Urgency of surgery is defined as *elective*: surgery that is scheduled in advance because it does not involve a medical emergency, *urgent*: surgery required within < 48 h*, emergent*: surgery performed when the patients’ life or well being are threatened

^d^Duration of surgery is the time between skin incision and closure of the incision

^e^Duration of anaesthesia is the time between start of induction and tracheal extubation or discharge from operation room if the mechanical ventilation is continued

^f^Daytime surgery is defined as anaesthesia induction between 8:00 a.m. and 19:59 p.m.

**Table 2:** Intraoperative ventilatory setting by group

	All patients (***N*** = 2034)	Closed abdominal surgery (N = 906)	Open abdominal surgery (***N*** = 1128)	***P–***value	Absolute Difference
**Ventilation mode, % (n/N)**				0.013	Pressure–controlled 4% [1 to 8%]
Volume–controlled	77% (1571/2034)	79% (895/906)	75% (676/1128)		
Pressure–controlled	23% (463/2034)	21% (233/906)	25% (230/1128)		
**Tidal Volume**					
Absolute (ml)	505 [465 to 570]	504 [462 to 570]	505 [465 to 572]	0.567	1 [24 to 25]
Per PBW (ml∙kg^− 1^)	8.0 [7.0 to 9.0]	8.5 [7.6 to 9.5]	8.2 [7.4 to 9.2]	0.001	0.2 [0.07 to 0.5]
Per ABW (ml∙kg^− 1^)	7.0 [6.0 to 8.0]	6.8 [5.8 to 7.7]	7.0 [6.1 to 7.9]	< 0.001	0.2 [0.1 to 0.4]
**Minute ventilation (L∙kg**^**− 1**^**)**	6.0 [6.0 to 7.0]	6.5 [5.8 to 7.2]	6.3 [5.5 to 7.0]	< 0.001	0.2 [0.1 to 0.4]
**Respiratory system compliance**					
Dynamic, ml∙cm∙H_2_O^−1^	26 [21 to 32]	25 [20 to 32]	27 [21 to 33]	< 0.001	2 [0 to 4]
Static, ml∙cm∙H_2_O^−1^	42 [35 to 50]	41 [33 to 50]	43 [36 to 51]	< 0.001	1 [0.4 to 2]
**Routine recruitment maneuvers, % (n/N)**	12% (238/2.029)	13% (119/905)	11% (119/1.124)	0.087	2% [1 to 5%]
**FiO**_**2**_**, %**	50 [45 to 56]	54 [48 to 70]	50 [45 to 63]	< 0.001	4 [4 to 10]
**SpO**_**2**_**, %**	99 [98 to 100]	99 [98 to 100]	99 [98 to 100]	< 0.001	0 [0 to 0]^a^
**EtCO**_**2**_**, kPa**	4.0 [4.0 to 5.0]	4.6 [4.2 to 4.9]	4.3 [4.0 to 4.7]	< 0.001	0.2 [0.2 to 0.6]
**Airway pressures**					
**Driving pressure**					
Time–weighted average (cmH_2_O∙hour^−1^)	8 [6 to 11]	8 [6 to 11]	8 [6 to 10]	0.091	0.2 [0.09 to 1.2]
Maximum value (cmH_2_O)	14 [11 to 18]	16 [12 to 20]	14 [11 to 17]	< 0.001	2 [2 to 7]
Minimum value (cmH_2_O)	11 [9 to 14]	11 [9 to 15]	11 [9 to 14]	0.008	0 [0 to 17]
Coefficient of variation (%)	10 [5 to 20]	15 [6 to 26]	9 [4 to 15]	< 0.001	5 [4 to 8]
**Peak pressure**					
Time–weighted average (cmH_2_O∙hour^−1^)	12 [9 to 15]	11 [9 to 15]	12 [9 to 15]	0.414	0.2 [0.1 to 1.1]
Highest value (cmH_2_O)	20 [17 to 24]	21 [18 to 26]	19 [16 to 23]	< 0.001	2 [2 to 10]
Lowest value (cmH_2_O)	16 [14 to 20]	17 [14. to 20]	16 [14 to 20]	0.011	1 [1 to 3]
Coefficient of variation (%)	8 [4 to 15]	11 [5 to 19]	7 [3 to 12]	< 0.001	5 [3 to 6]
**PEEP**					
Time–weighted average (cmH_2_O∙hour^−1^)	2 [1 to 3]	2 [1 to 4]	2 [1 to 3]	0.019	0 [0 to 0]
Highest value (cmH_2_O)	5 [2 to 5]	5 [2 to 5]	5 [2 to 5]	0.255	0 [0 to 0]
Lowest value (cmH_2_O)	4 [0 to 5]	4 [0 to 5]	3 [0 to 5]	0.186	1 [1 to 5]
Coefficient of variation (%)	0 [0 to 22]	0 [0 to 22]	0 [0 to 22]	0.579	0 [0 to 0]

Data are presented as median [25th–75th percentile] or % (n/N). For binary and continuous variables risk difference and median difference with confidence intervals are reported respectively. Abbreviations: *EtCO*_*2*_ End-tidal CO_2_, *FiO*_*2*_ Fraction of inspired oxygen, *SpO*_*2*_ Peripheral oxygen saturation, *OR* Odds ratio

^a^Difference between groups is significant but very small and masked by rounding process

### Primary and secondary outcome rates

In 102 (5%) patients, one or more PPC occurred, with a higher prevalence in open surgery patients than in patients who underwent a closed surgical procedure (7 versus 3%; *P* <  0.001). Hypotension, or need for vasopressors was more frequently observed during open surgery, while the need for airway pressure reduction was more often needed during closed surgery (Table 3).

**Table 3:** Intraoperative and postoperative outcomes

	All patients (N = 2.034)	Closed abdominal surgery (N = 906)	Open abdominal surgery (N = 1.128)	***P–*** value
**Severe PPC (composite), % (n/N)**	5% (102/2.034)	3% (28/906)	7% (74/1.128)	0.001
**Intraoperative complications**				
Desaturation	4% (73/2.026)	3% (26/903)	4% (47/1.123)	0.148
Unplanned rescue maneuvers	4% (87/2.026)	4% (41/903)	4% (46/1.123)	0.704
Need for ventilatory pressure reduction	4% (77/2.025)	6% (57/903)	2% (20/1.102)	< 0.001
Expiratory flow limitation	1% (14/2.015)	1% (12/898)	0% (2/1.117)	0.005
Hypotension	28% (562/2.027)	20% (182/903)	34% (380/1.124)	< 0.001
Use of vasopressors	23% (469/2.027)	17% (153/903)	28% (316/1.122)	< 0.001
New arrhythmia onset	1% (13/2.027)	0% (2/903)	1% (11/1.124)	0.065
**Individual PCCs**				
Acute respiratory failure	3% (58/2.034)	2% (21/906)	3% (37/1.128)	0.245
Need for mechanical ventilation	2% (44/2.034)	1% (11/906)	3% (33/1.128)	0.013
Acute respiratory distress syndrome	0% (6/2.034)	0% (0/906)	0% (6/1.128)	0.074
Pneumonia	0% (16/2.034)	0% (2/906)	1% (14/1.128)	0.019
Pneumothorax	0% (4/2.034)	0% (0/906)	0% (4/1.128)	0.186
**In–hospital mortality**	1% (22/1.892)	0% (3/838)	2% (19/1.054)	0.007
**Length of stay** (days)	3 [1 to 5]	1 [0 to 3]	5 [2 to 8]	< 0.001

Data are presented as median [25th–75th percentile] or % (n/N)

*PPC* Postoperative pulmonary complications

### Propensity score estimation variables

The variables that finally entered the propensity score and covariate balance assessment are detailed in the Additional file 2: Statistics and Fig. S2 and S3.

### Association of ΔP_TW_ with PPCs

ΔP_TW_ was significantly associated with PPCs in both surgical groups. The association was stronger in closed abdominal surgery patients (odds ratio (OR), 1.17 [95%CI 1.16 to 1.19]; *P* <  0.001; risk ratio (RR), 1.11 [95%CI 1.10 to 1.20], *P* <  0.001) than in patients who underwent an open abdominal surgical intervention (OR, 1.07 [95%CI 1.06 to 1.08]; *P* <  0.001; RR 1.05 [95% CI 1.05 to 1.05]), with a significant difference (difference between ORs: 0.09 [95%CI 0.07 to 0.10]; *P* <  0.001; risk difference 0.05: [95%CI 0.04 to 0,06]), *P* <  0.001. Residuals plots are reported in Additional file 2: Figure S4.

### Association of ΔP_TW_ with the occurrence of adverse events

ΔP_TW_ was significantly associated with intraoperative adverse events in both open and closed surgery patients. Also, here the association was stronger in closed surgery patients (1.13 [95%CI 1.12 to 1.14]) than in patients who underwent an open abdominal intervention (1.07 [95%CI 1.05 to 1.10]), difference between ORs 0.05 [95%CI 0.03 to 0.07]; *P* <  0.001.

### Sensitivity analyses

**Fig. 3:**
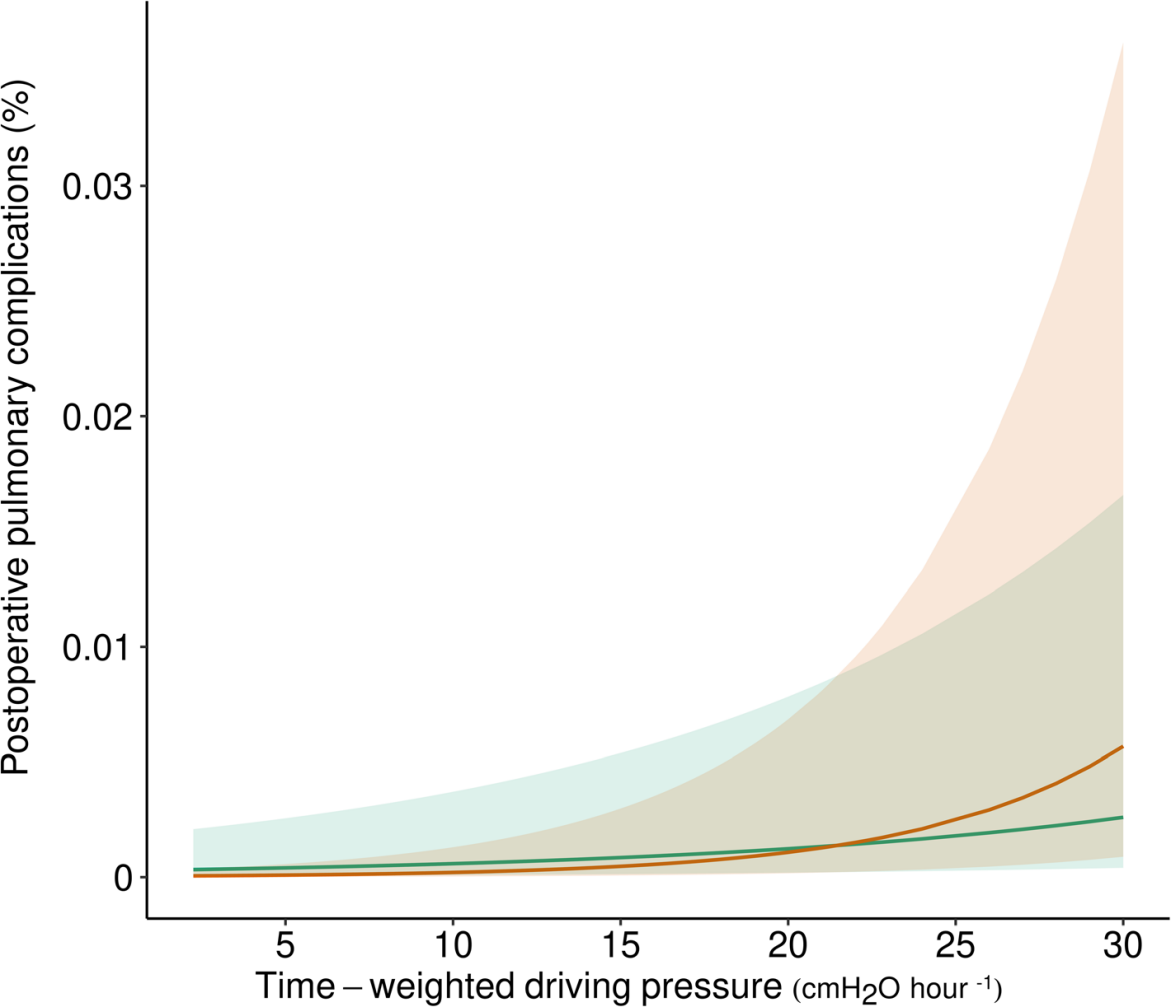
Marginal effect plot of time–weighted average driving pressure on the probability of postoperative pulmonary complications by type of surgery. Green: open surgery, Orange: closed surgery; solid lines are estimated marginal mean effect, and bandwidths are 95% confidence intervals

ΔP_TW_ was significantly associated with PPCs (OR, 1.08 [95%CI 1.06 to 1.09], *P* <  0.001) with closed surgery patients having a lower probability of occurrence (OR, 0.14 [95%CI 0.12 to 0.16, *P* <  0.001) with a significant interaction between ΔP_TW_ and closed surgery (OR, 1.09 [95%CI 1.08 to 1.11], *P* <  0.001). The marginal effect of ΔP_TW_ by type of surgery on PPCs probability is showed in Fig. 3**.** A rise in ΔP_TW_ was associated with an increased probability of PPCs in both surgery types, with a steeper increase in closed surgery patients for ΔP_TW_ above 20 cmH_2_O ∙ hour^− 1^.

After matching, the resulting cohort consisted of 344 open surgery patients and 254 closed surgery patients. Baseline characteristics between groups were well balanced (Additional file 2: Table S4 and S5). Type of surgery at matched levels of driving pressure was not associated with either outcome. (Additional file 2: Table S5 and S6).

## Discussion

The main findings of this posthoc analysis of the LAS VEGAS study can be summarised as follows: (i.) the intraoperative ΔP_TW_ was not different between open and closed surgery groups; (ii.) ΔP_TW_ was associated with PPCs in both closed and open surgery patients; (iii.) ΔP_TW_ was associated with intraoperative adverse events in both closed and open surgery patients; and (iv.) the type of surgery had a modifying effect on the association between **ΔP**_**TW**_ and PPCs, with an increasing probability of PPCs at high ΔP_TW_ in closed surgery. The last finding, though, was not confirmed in the matched cohort analysis.

This analysis uses the database of a worldwide international multicentre prospective observational study as a convenience sample [10], strictly followed a plan, and was characterised by a robust method accounting for the multilevel data structure and allowing precise estimation and confounder control, even with seven or fewer events per confounder [17, 18]. Also, the outcome of interest, i.e., PPCs, was predefined, well–described, and largely followed the European Perioperative Clinical Outcome (EPCO) group definitions [19]. Furthermore, the study population was defined to minimise information and selection bias and to have a sufficient number of patients while keeping an acceptable number of timepoints at which **ΔP**_**TW**_ could be calculated per patient.

A recent metanalysis of individual trials on protective ventilation during general anaesthesia for cardiac or thoracic surgery found a significant association between **ΔP**_**TW**_ and PPCs (OR 1.16, 95% CI 1.13 to 1.19; *p* <  0·0001) [5]. We found an almost identical association in patients undergoing closed abdominal surgery. Thus, our results confirm that **ΔP**_**TW**_ is a promising target for interventions to prevent PPCs after closed abdominal surgery. The sensitivity analysis showed that the association between **ΔP**_**TW**_ and PPCs was lower in patients who underwent a closed surgical procedure. However, this was not confirmed in the propensity score matched analysis, probably because of smaller sample size due to the matching procedure.

ΔP is an indicator of the amount of strain delivered to the respiratory system during mechanical ventilation [7]. Several studies investigated the effect of pneumoperitoneum on respiratory mechanics. Pneumoperitoneum was consistently found to decrease chest wall compliance, whereas lung compliance seems mostly spared by it [20–27]. Thus, inferring the amount of lung strain from plateau pressure and PEEP during pneumoperitoneum is challenging, since the part of the rise in plateau pressure caused by chest wall stiffening should not be regarded as a rise in lung strain [28]. Consequently, a higher ΔP during closed abdominal surgery is often seen as innocent. The current analysis results reject this assumption, as the association of ΔP with PPCs was stronger in patients undergoing closed abdominal surgery than in patients undergoing open abdominal surgery.

Pneumoperitoneum can affect lung mechanics in several ways [20–27]. A cranial shift of the diaphragm during laparoscopic surgery increases alveolar collapse, especially in lung parts close to the diaphragm. This is particularly true in upper abdominal surgery, which was the most common surgical procedure in patients undergoing closed surgery in the here studied cohort [29, 30]. PEEP may partially prevent this, and usually only when using high PEEP [31]. In the patients studied here, mostly low PEEP was used, regardless of the group. Additional studies are needed to test how high PEEP affects the association between ΔP with PPCs during pneumoperitoneum. Also, we found that ΔP was higher in patients undergoing closed surgery than in patients undergoing open abdominal surgery. However, open abdominal surgery lasted longer, resulting in a comparable **ΔP**_**TW**_ in the two groups. The higher absolute ΔP was compensated for by a shorter duration of intraoperative ventilation, and vice versa. Using the **ΔP**_**TW**_ allowed us to estimate an exposure limit threshold to an injurious factor as in occupational health. The steeper increase in probability of PPCs above a 20 cm H_2_O∙hour^− 1^ found in the sensitivity analysis can be related to an increase in collapsed lung tissue.

As expected, PPCs occurred more frequently in open abdominal surgery patients. An increased baseline risk could explain this due to typical differences in patient characteristics and the duration and the type of surgery. However, this finding strengthens the current analysis since we observed the association even in a cohort of patients, i.e., closed abdominal surgery, at low risk for PPCs and even after controlling for confounding effects with propensity score analysis.

Several intraoperative ventilation approaches, like the use of recruitment manoeuvres and higher PEEP, may result in a lower ΔP [32, 33]. Findings of a metanalysis including clinical trials on intraoperative ventilation suggest that PEEP titrations that resulted in a ΔP rise increased the risk of PPCs [5]. One randomised clinical trial showed an intraoperative PEEP strategy targeting the best compliance to reduce PPCs, though this was only a secondary endpoint in that study [34]. Thus, the best approach to minimise PPCs remains a matter of debate.

**ΔP**_**TW**_ was associated with intraoperative adverse events in both closed and open surgery patients. Among all adverse events, airway pressure reduction was more frequently needed in closed surgery group underlining the need for ventilation strategies to lower peak and plateau pressures in this group of patients reflecting unacceptable high airway pressure during surgery.

Several limitations must be acknowledged. We used the parent LAS VEGAS definition of PPCs. This definition differs from what was somewhat recently proposed [1], but they remain reasonably comparable. The protocol of the LAS VEGAS study did not include the collection of oesophageal pressure recordings. Information regarding surgical positioning was not collected, and intra–abdominal pressure levels were also not recorded in the database of the LAS VEGAS study. Both could influence **ΔP**_**TW**_, though [35–37]. Due to the additional strict exclusion criteria, we excluded a considerable number of patients. Thus, the findings of this analysis need confirmation in other studies. Also, some patients had only a few timepoints at which ΔP could be calculated. Furthermore, we only included patients with an endotracheal tube and patients who received controlled ventilation, limiting our focus on a specific type of intraoperative airway device and ventilation mode. Of note, 25% of patients had a Body Mass Index (BMI) > 30 kg∙m^− 2^. Extrapolating this analysis’s findings to obese or morbidly obese patients should be done with some caution. Also, the original LAS VEGAS study was performed 7 years ago. Since then, there could have been changes in clinical practice, e.g., in the use of ‘Enhanced Recovery After Surgery’ (ERAS) pathways and muscle relaxant monitoring during and reversal at the end of surgery. Although the time gap between research findings and practice changes usually lasts longer than a decade [38–40], still could be that more immediate changes may affect the associations. Finally, we did not set any a priori effect threshold nor multiple comparisons correction; hence the results’ statistical significance and the exploratory nature of secondary outcome analysis must be confirmed in future trials.

## Conclusions

ΔP_TW_ is associated with the occurrence of PPCs and intraoperative adverse events in abdominal surgery. These associations are present regardless of the type of surgical approach and depend on the duration and actual ΔP. Both in patients undergoing open or closed abdominal surgery, the ΔP is a promising target for future strategies to **reduce** PPCs.

## Supplementary Information

**Additional file 1: Table 1.** Patient and surgery related characteristics. **Table 2.** Intraoperative venitlatory setting by group.

**Additional file 2: Table S1.** Definition of postoperative pulmonary complications. **Table S2**. Definition of intraoperative complications. **Table S3**. Number of data available at each time point. **Table S4**. Patients demographics and surgery–related characteristics in the matched cohort for type of surgery. **Table S5**. Intraoperative and postoperative outcomes in matched cohort for type of surgery. **Table S6**. Mixed multivariable logistic regression in matched cohort for postoperative pulmonary complications. **Figure S1.** Time weighted average and coefficient of variation calculation. **Figure S2.** Summary plot of covariate balance for time-weighted ΔP before (red line) and after (blue line) conditioning for open surgery. **Figure S3.** Summary plot of covariate balance before (red line) and after (blue line) conditioning for closed surgery. **A**: time–weighted; **B**: Highest value; **C:** Lowest Value; **D**: Coefficient of variation. **Figure S4.** Residuals plot for postoperative pulmonary complications (PPCs) and intraoperative adverse events (AEs). A: PPCs in Open surgery; B: PPCs in closed surgery; C; AEs in open surgery; D: AEs in closed surgery.

## Data Availability

The data as well as the code used for analysis are available from the corresponding author upon reasonable request.

## Abbreviations

ΔP: Driving pressure
ΔP_TW_: Time–weighted average ΔP
V_T_: Tidal volume
PEEP: Positive end–expiratory pressure
STROBE: Strengthening the reporting of observational studies in epidemiology
ARISCAT: Assess Respiratory Risk in Surgical Patients in Catalonia
AUC: Area Under Curve
RR: Risk ratio
OR: Odds ratio
BMI: Body Mass Index
EPCO: European Perioperative Clinical Outcome
ERAS: Enhanced Recovery After Surgery’
