# Supplementary material for: The Association of Intraoperative driving pressure with postoperative pulmonary complications in open versus closed abdominal surgery patients – a posthoc propensity score–weighted cohort analysis of the LAS VEGAS study
Source: BMC Anesthesiol. 2021 Mar 19;21:84. doi: 10.1186/s12871-021-01268-y (PMC7977277; doi:10.1186/s12871-021-01268-y)
Supplement: Supplementary file 1 — Additional file 1: Table 1. Patient and surgery related characteristics. Table 2. Intraoperative venitlatory setting by group. [file 12871_2021_1268_MOESM1_ESM.pdf]

Valencia January 3, 2019

LAS VEGAS secondary analysis in driving pressure, Analysis plan outline

General description of statistical methods

Normally distributed variables will be expressed by their mean and standard deviation; not normally distributed variables will be expressed by their median and interquartile ranges; categorical variables will be expressed as n (%). Normality of distributions will be assessed by inspecting quantile–quantile plots. In test groups of continuous normally distributed variables, Student's t-test will be used. Likewise, if continuous data are not normally distributed the Wilcoxon rank sum test will be used. Categorical variables will be compared with the Chi-square test or Fisher's exact test or when appropriate as relative risks. Statistical uncertainty will be expressed by 95% confidence levels.

Dicothomic outcomes will be analyzed by mixed logistic regression while cumulative variable will be analyzed with by mixed ordinal regression. All models will be fitted with a weighting factor using an inverse probability weighting factor to control for confounders and including a random effect for center.

All analyses will be performed with R 3.5.1 (The R Foundation for Statistical Computing, www.r-project.org). Multiple imputation of missing values will be considered when appropriate. No correction for multiple comparisons is prespecified, thus, all the findings should be viewed as exploratory. A P–value of less than 0.05 will be considered statistically significant.

Baseline descriptive statistics and variables definition

Baseline characteristics will be presented as shown in **dummy Table 1 and 2.** Patients will be grouped according to the type if surgery performed (open vs. closed).

Driving pressure (ΔP) will be calculated by subtracting PEEP from Pplat in volume–controlled ventilation, and by subtracting PEEP from Pmax in pressure–controlled ventilation. For intraoperative ventilatory related variables that are hourly–collected during surgery (from induction to end of anesthesia up to the 7th hour of surgery), a time weighted average value will be calculated for every participant for these variables for analysis. The highest and lowest measured ΔP (ΔP_{HI} and ΔP_{LO}) will be collected for each patient. The ΔP coefficient of variation (ΔP_{CV}), as a measure of ΔP variability, will be calculated by dividing the standard deviation (SD) by the mean ΔP level. As for ΔP, the time–weighted average P_{peak} and PEEP, highest and lowest measured P_{peak} and PEEP, and P_{peak} and PEEP coefficients of variation will be determined. For other ventilatory parameters median values will be collected.

Analysis of the primary outcome

The primary outcome of this analysis are sever PPCs as defined in the original LAS VEGAS protocol. Severe PPCs is a composite outcome that comprises: unexpected postoperative invasive or non–invasive ventilation, acute respiratory failure, acute respiratory distress syndrome, pneumonia, and pneumothorax. The occurrence of each type of PPC was monitored until hospital discharge, but restricted to the first five postoperative days.

To determine whether driving pressure is associated with development of PPCs we will build a mixed logistic regression model for closed and open surgery cohort respectively and including a random factor for center. To fit the model a weight score will be built based on the covariate-balancing propensity score (CBPS) methodology to simultaneously optimize prediction of treatment assignment and confounders influence. To build the propensity score we will include the following variables: ARISCAT class and intraoperative tidal volume (V_T). In addition to these variables included by clinical judgement, we will refine covariate entry with a augmented backward elimination selection process. All variables enter the model and finally those pre– and intraoperative factors that yielded a change in the effect estimate > 0.1 and with and a significance criterion (alpha) < 0.1 will be included for CBPS estimation. Odds ratios will be compared with a Wald z–test.

Analysis of the secondary outcomes

A composite of prespecified intraoperative events will be analyzed as secondary endpoint. This composite outcome comprises: 1) intraoperative desaturation (SpO_2 < 92% for more than 2 minutes); 2) need for unplanned lung recruitment maneuvers (defined as ventilator strategies aimed to restore aeration of the lungs); 3) hypotension (systolic arterial blood pressure < 90 mmHg for 3 minutes or longer); 4) use of vasoactive drugs to correct hypotension; 5) new onset of expiratory flow limitation (expiratory flow higher than zero at end expiration as suggested by visual analysis of flow 1 curve); 6) need for ventilator pressure reduction (defined as ventilatory strategies aimed to lower peak and

plateau pressure); and 7) onset of new arrhythmias (atrial fibrillation, sustained ventricular tachycardia, supraventricular tachycardia, or ventricular fibrillation).

To determine whether driving pressure is associated with development of intraoperative adverse events we build a Bayesian ordinal regression model for closed and open surgery cohort respectively and including a random factor for center. The model will be fit with an inverse probability weighting with an estimator obtained with CBPS calculation as for the primary outcome.

TABLE 1 . Patient and surgery related characteristics

	All patients (n=)	Laparoscopic Surgery (n=)	Open surgery (n=)	*P* value	Standardized Difference
**Age (years)**	mean±SD /median [IQR]	mean±SD /median [IQR]	mean±SD /median [IQR]		
**Gender (Male/Female)**	%/% (n/n)	%/% (n/n)	%/% (n/n)		
**Ethnicity**					
Caucasian	% (n/total)	% (n/total)	% (n/total)		
Black	% (n/total)	% (n/total)	% (n/total)		
Asian	% (n/total)	% (n/total)	% (n/total)		
Other	% (n/total)	% (n/total)	% (n/total)		
**BMI ($Kg\cdot m^{-2}$)**	mean±SD /median [IQR]	mean±SD /median [IQR]	mean±SD /median [IQR]		
**Weight (kg)**	mean±SD /median [IQR]	mean±SD /median [IQR]	mean±SD /median [IQR]		
**PBW (kg)**	mean±SD /median [IQR]	mean±SD /median [IQR]	mean±SD /median [IQR]		
**ASA class**					
1	% (n/total)	% (n/total)	% (n/total)		
2	% (n/total)	% (n/total)	% (n/total)		
3	% (n/total)	% (n/total)	% (n/total)		
4	% (n/total)	% (n/total)	% (n/total)		
5	% (n/total)	% (n/total)	% (n/total)		
**ARISCAT score**	mean±SD /median [IQR]	mean±SD /median [IQR]	mean±SD /median [IQR]		
**ARISCAT class**					
< 26	% (n/total)	% (n/total)	% (n/total)		
26 – 44	% (n/total)	% (n/total)	% (n/total)		
> 44	% (n/total)	% (n/total)	% (n/total)		
**Preoperative SpO_2 (%)**	mean±SD /median [IQR]	mean±SD /median [IQR]	mean±SD /median [IQR]		
**Current smoker**	% (n/total)	% (n/total)	% (n/total)		
**Chronic co-morbidity**					
Metastatic cancer	% (n/total)	% (n/total)	% (n/total)		
Chronic kidney failure	% (n/total)	% (n/total)	% (n/total)		
COPD	% (n/total)	% (n/total)	% (n/total)		
Heart failure	% (n/total)	% (n/total)	% (n/total)		
Obstructive sleep apnoeas	% (n/total)	% (n/total)	% (n/total)		
Neuromuscular disease[a]	% (n/total)	% (n/total)	% (n/total)		
Liver dysfunction	% (n/total)	% (n/total)	% (n/total)		
Circulation	% (n/total)	% (n/total)	% (n/total)		
Neurology	% (n/total)	% (n/total)	% (n/total)		
Renal	% (n/total)	% (n/total)	% (n/total)		
**Active Smoker**	% (n/total)	% (n/total)	% (n/total)		
**Preoperative SpO_2 (%)**	mean±SD /median [IQR]	mean±SD /median [IQR]	mean±SD /median [IQR]		
**Respiratory infection < 30 days prior to surgery**	% (n/total)	% (n/total)	% (n/total)		
**Preoperative Hb ($g\cdot dl^{-1}$)**	mean±SD /median [IQR]	mean±SD /median [IQR]	mean±SD /median [IQR]		
**Preoperative anemia (Hb ≤ 10 g dl^{-1})**	% (n/total)	% (n/total)	% (n/total)		
**RBC Transfusion < 30 days**	% (n/total)	% (n/total)	% (n/total)		
**Surgical procedure**					
Lower GI	% (n/total)	% (n/total)	% (n/total)		
Upper GI, Hepatobiliopancreatic	% (n/total)	% (n/total)	% (n/total)		
Vascular surgery	% (n/total)	% (n/total)	% (n/total)		
Aortic surgery	% (n/total)	% (n/total)	% (n/total)		
Urological	% (n/total)	% (n/total)	% (n/total)		
Gynaecological	% (n/total)	% (n/total)	% (n/total)		
Endocrine surgery	% (n/total)	% (n/total)	% (n/total)		
Transplant	% (n/total)	% (n/total)	% (n/total)		
Other procedure	% (n/total)	% (n/total)	% (n/total)		
**Urgency of Surgery**					
Elective	% (n/total)	% (n/total)	% (n/total)		
Urgent	% (n/total)	% (n/total)	% (n/total)		

Emergency	% (n/total)	% (n/total)	% (n/total)
**Duration of surgery[c] (min)**	mean±SD /median [IQR]	mean±SD /median [IQR]	mean±SD /median [IQR]
**Duration of anaesthesia[d] (min)**	mean±SD /median [IQR]	mean±SD /median [IQR]	mean±SD /median [IQR]
**Antibiotic prophylaxis**	mean±SD /median [IQR]	mean±SD /median [IQR]	mean±SD /median [IQR]
**RBC Transfusion during surgery**	% (n/total)	% (n/total)	% (n/total)
**Units of RBC Transfused**	mean±SD /median [IQR]	mean±SD /median [IQR]	mean±SD /median [IQR]
**Anaesthesia technique**			
Epidural anaesthesia	% (n/total)	% (n/total)	% (n/total)
**Opioid**			
Short-acting	% (n/total)	% (n/total)	% (n/total)
Long-acting	% (n/total)	% (n/total)	% (n/total)
Both long- and short-acting	% (n/total)	% (n/total)	% (n/total)
Neuromuscular Blockade	% (n/total)	% (n/total)	% (n/total)
Neuromuscular Monitoring	% (n/total)	% (n/total)	% (n/total)
Neuromuscular Reversal	% (n/total)	% (n/total)	% (n/total)
TIVA	% (n/total)	% (n/total)	% (n/total)
Total Fluids ($mL\cdot kg^{-1}$)	mean±SD /median [IQR]	mean±SD /median [IQR]	mean±SD /median [IQR]
Crystalloids ($mL\cdot kg^{-1}$)	mean±SD /median [IQR]	mean±SD /median [IQR]	mean±SD /median [IQR]
Colloids ($mL\cdot kg^{-1}$)	mean±SD /median [IQR]	mean±SD /median [IQR]	mean±SD /median [IQR]

Data are presented as median [IQR] or % (numebr of event/total of sample).
BMI: body mass index; ASA: American Society of Anesthesiologists; Hb: haemoglobin; GI: gastrointestinal; SpO2: peripheral oxygen saturation: RBC: Red Blood cells.
[a]Neuromuscolar disease affecting the respiratory system.
[b] Urgency of surgery: elective: surgery that is scheduled in advance because it does not involve a medical emergency, urgent: surgery required within <48 hours, emergency: surgery performed when the patients life or well being is threatened.
[c]Duration of surgery is the time between skin incision and closure of the incision.
[d]Duration of anaesthesia is the time between start of induction and tracheal extubation or discharge from operation room if the mechanical ventilation continued.

TABLE 2 . Intraoperative venitlatory setting by group

	All patients (n=)	Laparoscopic Surgery (n=)	Open surgery (n=)	*P* value	Standardized Difference
**Ventilation mode**					
Volume–controlled	% (n/total)	% (n/total)	% (n/total)		
Pressure–controlled	% (n/total)	% (n/total)	% (n/total)		
**Tidal Volume**					
Absolute (ml)	median [IQR]	median [IQR]	median [IQR]		
Per PBW (ml·kg^{-1})	median [IQR]	median [IQR]	median [IQR]		
Per actual BW (ml·kg^{-1})	median [IQR]	median [IQR]	median [IQR]		
**Minute ventilation (L·kg^{-1})**	median [IQR]	median [IQR]	median [IQR]		
**Compliance of the respiratory system**	median [IQR]	median [IQR]	median [IQR]		
Dynamic (ml·cm·H2O^{-1})	median [IQR]	median [IQR]	median [IQR]		
Static (ml·cm·H2O^{-1})	median [IQR]	median [IQR]	median [IQR]		
**Routine recruitment maneuvers**					
Not performed	median [IQR]	median [IQR]	median [IQR]		
Bag squeezing	median [IQR]	median [IQR]	median [IQR]		
Ventilator	median [IQR]	median [IQR]	median [IQR]		
**FiO_2 (%)**	median [IQR]	median [IQR]	median [IQR]		
**SpO_2 (%)**	median [IQR]	median [IQR]	median [IQR]		
**$EtCO_2$ (%)**	median [IQR]	median [IQR]	median [IQR]		
**Ventilatory frequency (cycles min^{-1})**	Time weighted mean±SD	Time weighted mean±SD	Time weighted mean±SD		
**Airway pressures**					
PEEP (cm·H_20)	Time weighted mean±SD	Time weighted mean±SD	Time weighted mean±SD		
Peak (cm·H_20)	Time weighted mean±SD	Time weighted mean±SD	Time weighted mean±SD		
