# Supplementary material for: The Association of Intraoperative driving pressure with postoperative pulmonary complications in open versus closed abdominal surgery patients – a posthoc propensity score–weighted cohort analysis of the LAS VEGAS study
Source: BMC Anesthesiol. 2021 Mar 19;21:84. doi: 10.1186/s12871-021-01268-y (PMC7977277; doi:10.1186/s12871-021-01268-y)
Supplement: Supplementary file 2 — Additional file 2: Table S1. Definition of postoperative pulmonary complications. Table S2. Definition of intraoperative complications. Table S3. Number of data available at each time point. Table S4. Patients demographics and surgery–related characteristics in the matched cohort for type of surgery. Table S5. Intraoperative and postoperative outcomes in matched cohort for type of surgery. Table S6. Mixed multivariable logistic regression in matched cohort for postoperative pulmonary complications. Figure S1. Time weighted average and coefficient of variation calculation. Figure S2. Summary plot of covariate balance for time-weighted ΔP before (red line) and after (blue line) conditioning for open surgery. Figure S3. Summary plot of covariate balance before (red line) and after (blue line) conditioning for closed surgery. A: time–weighted; B: Highest value; C: Lowest Value; D: Coefficient of variation. Figure S4. Residuals plot for postoperative pulmonary complications (PPCs) and intraoperative adverse events (AEs). A: PPCs in Open surgery; B: PPCs in closed surgery; C; AEs in open surgery; D: AEs in closed surgery. [file 12871_2021_1268_MOESM2_ESM.docx]

SUPPLEMENTARY DIGITAL CONTENT

**The Association of Intraoperative Driving Pressure with Postoperative Pulmonary Complications in Open versus Closed Abdominal Surgery Patients – a posthoc propensity score–weighted cohort analysis of the LAS VEGAS study**

Guido Mazzinari^1,2,*^ M.D. Ph.D. M.Sc., Ary Serpa Neto^3,4,5^ M.D. Ph.D. M.Sc., Sabrine NT. Hemmes^5^ M.D. Ph.D., Goran Hedenstierna^6^ M.D. Ph.D., Samir Jaber^7^ M.D. Ph.D., Michael Hiesmayr^8^ M.D. Ph.D., Markus W. Hollmann^5^ M.D. Ph.D., Gary H. Mills^9^ M.D., Marcos F. Vidal Melo^10^ M.D. Ph.D., Rupert M. Pearse^11^ M.D. Ph.D., Christian Putensen^12^ M.D., Werner Schmid^8^ M.D., Paolo Severgnini^13^ M.D., Hermann Wrigge^14^ M.D., Oscar Diaz–Cambronero^1,2^ M.D., Lorenzo Ball^15,16^ M.D. Ph.D., Marcelo Gama de Abreu^17^ M.D. Ph.D., Paolo Pelosi^15,16^  M.D. FERS and Marcus J. Schultz^5,18,19^ M.D. Ph.D. for the LAS VEGAS* study–investigators**, the PROtective VEntilation NETwork, and the Clinical Trial Network of the European Society of Anesthesiology

*‘Local Assessment of VEntilatory management during General Anesthesia for Surgery’; **a complete list of the LAS VEGAS study–collaborators is provided in the Appendix

**Hospital Universitario y Politécnico la Fe, Valencia, Spain**

^1^Research Group in Perioperative Medicine; ^2^Department of Anesthesiology;

**Hospital Israelita Albert Einstein, São Paulo, Brazil**

^3^Department of Critical Care Medicine

**Instituto do Coração, Hospital das Clinicas HCFMUSP, Faculdade de Medicina, Universidade de Sao Paulo, Sao Paulo, Brazil**

^4^Cardio-Pulmonary Department, Pulmonary Division

**Academic Medical Center, Amsterdam, The Netherlands**

^5^Department of Intensive Care & Laboratory of Experimental Intensive Care and Anesthesiology (L·E·I·C·A)

**Uppsala University, Sweden**

^6^Department of Medical Sciences, Clinical Physiology
**University of Montpellier, France**

^7^PhyMedExp, INSERM U1046, CNRS UMR 9214

**Medical University Vienna, Vienna, Austria**

^8^Division Cardiac, Thoracic, Vascular Anesthesia and Intensive Care

**Sheffield Teaching Hospitals, Sheffield and University of Sheffield, United**

**Kingdom**

^9^Operating Services, Critical Care and Anesthesia

**Massachusetts General Hospital, Boston, U.S.A.**

^10^Department of Anesthesia, Critical Care and Pain Medicine

^11^**Queen Mary University of London, London, UK**

**University Hospital Bonn, Bonn, Germany**

^12^Department of Anesthesiology and Intensive Care Medicine

**University of Insubria, Varese, Italy**

^13^Department of Biotechnology and Sciences of Life, ASST- Settelaghi Ospedale di Circolo e Fondazione Macchi

**University of Leipzig, Leipzig, Germany**

^14^Department of Anesthesiology and Intensive Care Medicine

^15^**Policlinico San Martino Hospital – IRCCS for Oncology and Neurosciences, Genoa, Italy**

**University of Genoa Italy**

^16^Department of Surgical Sciences and Integrated Diagnostics

**Technische Universität Dresden, Dresden, Germany**

^17^Department of Anesthesiology and Intensive Care Therapy, Pulmonary Engineering Group

**Mahidol University, Bangkok, Thailand**

^18^Mahidol–Oxford Tropical Medicine Research Unit (MORU)

**University of Oxford, Oxford, UK**

^19^Nuffield Department of Medicine

Table of Contents

**Statistics – Details on covariate balancing propensity score calculation, variable selection process and propensity score matched posthoc analysis. 3**

**Supplementary Tables4**

**Table S1 –** Definition of postoperative pulmonary complications 4

**Table S2 –** Definition of intraoperative adverse events 5

**Table S3 –** Number of data available at each time point 6

**Table S4 –** Patients demographics and surgery–related characteristics in the matched cohort for type of surgery7

**Table S5 –** Intraoperative and postoperative outcomes in matched cohort for type of surgery 9

**Table S6 –** Mixed multivariable logistic regression in matched cohort for postoperative pulmonary complications 10

**Supplementary Figures11**

**Figure S1 –** Details on driving pressure measures calculations11

**Figure S2 –** Summary plot of covariate balancing score adjustment for each driving pressure measure in open surgery12

**Figure S3 –** Summary plot of covariate balancing score adjustment for each driving pressure measure in closed surgery13

**Figure S4** – Residuals plot for primary and secondary outcomes14

**References15**

**Statistics – General explanations and details on covariate balancing propensity score calculation, variable selection process and propensity score matched posthoc analysis.**

*Analysis plan roadmap*

We used a propensity score as the method of confounding bias control. The propensity score was estimated by using the covariate balancing method. The variables that entered the propensity score calculation were selected with a mixed strategy of prior clinical knowledge and feature selection. The propensity score was used in the main analysis as a weighting factor; this technique allows to retain all the information and thus the sample statistical power. The association between the main independent variable (time weighted driving pressure as a continuous variable) and primary outcome (postoperative pulmonary complication as a composite binary variable) was tested by fitting a weighted mixed effect logistic regression model and a random intercept by centre.

The variable selection process to enter the propensity score calculation was done using the augmented backwards elimination process implemented in the *abe* R package.^1^ The variables that finally entered the propensity score calculation for open surgery were: age, gender, body mass index, ASA risk score, functional status, smoker status, baseline comorbidities (chronic obstructive pulmonary disease, oncologic disease, heart failure, chronic kidney disease, obstructive sleep apnea disease, neurologic disease), duration of anesthesia, ARISCAT score (as individual variables) , type of surgery, urgency of surgery, time of surgery, previous and perioperative blood cell transfusion, use of epidural anesthesia, use of recruiting maneuvers, use of neuromuscular monitoring, use of neuromuscular blocking agents reversal, total fluid administration (body weight normalized), total crystalloid administration (body weight corrected), mode of mechanical ventilation, type of anesthetic agent (intravenous vs. halogenated), perioperative mechanical ventilation characteristics (median minute ventilation, median respiratory rate, median fraction of inspired oxygen, median end–tidal CO_2_ and median SpO2, median per–actual body weight V_T_, median static and dynamic compliance, highest PEEP, lowest PEEP and PEEP coefficient of variation, and driving pressure measures other than the main dependent variable analyzed). The variables that finally entered the propensity score calculation for closed surgery were the same with the addition of antibiotic prophylaxis.

For secondary endpoint (association between driving pressure and intraoperative adverse events) a weighted ordinal regression was fitted with *ordinal* R package. We chose this approach to better model the intraoperative adverse events variables as an ordinal variable where having two events in worse than one, three is worse than two etc. Weighting factor was the propensity score as described in the previous model for PPCs.

The propensity score for posthoc matched analysis was calculated with the same covariable structure as the CBPS with the *matchit* package.^2^

| **Table S1. Definition of postoperative pulmonary complications** | |
| --- | --- |
| **Single PPC** | **Definition** |
| **Unplanned need for oxygen therapy** | Supplemental O_2_ therapy administered to correct hypoxemia, defined as SpO_2_ < 90% in room air or PaO2 < 60 mmHg. This excludes oxygen administration given as a part of standard care, such as routine oxygen administration at the arrival in the PACU. |
| **Respiratory failure** | SpO_2_ < 90% in room air or PaO2 < 60 mmHg with oxygen therapy, or need for non-invasive mechanical ventilation |
| **Mechanical ventilation** | Need for new invasive ventilation after surgery, or unexpected prolonged invasive ventilation after discharge from the operating room |
| **ARDS** | According to the Berlin definition |
| **Pneumonia** | Lung infiltrates at the chest X-ray or CT, plus at least two among the following three criteria: fever > 38°C (100.4 °F), leucocytosis or leukopenia (WBC count > 12000 or <4000 cells/mm^3^), purulent secretions |
| **Pneumothorax** | Air in the pleural space without blood presence, as confirmed by chest X-ray |

***Composite outcomes***

**Severe PPC:** at least one PPC among respiratory failure, mechanical ventilation, ARDS, pneumonia, pneumothorax, as defined in eTable1.

| **Table S2. Definition of intraoperative complications** | |
| --- | --- |
| **Intraoperative Complication** | **Definition** |
| **De-saturation** | SpO_2_ < 92% |
| **Need for rescue recruitment manoeuvre** | Ventilation strategies aimed at restoring aeration of the lungs |
| **Need for ventilatory pressure reduction** | Ventilation strategies aimed at lowering peak and/or plateau pressure |
| **New onset of expiratory flow limitation** | End-expiration expiratory flow higher than zero at the visual analysis of the flow curve |
| **Hypotension** | Systolic arterial pressure < 90 mmHg for at least 3 minutes |
| **Need for vasoactive drugs** | Use of vasoactive drugs to correct hypotension as previously defined |
| **Arrhythmia** | Defined as any new onset of atrial fibrillation, sustained ventricular tachycardia, supraventricular tachycardia, or ventricular fibrillation |

| **Table S3. Number of data available at each time point.** | | | | | | | | |
| --- | --- | --- | --- | --- | --- | --- | --- | --- |
| **Time (hour)** | **0** | **1** | **2** | **3** | **4** | **5** | **6** | **7** |
| **Closed Surgery** | 775 | 906 | 433 | 175 | 78 | 30 | 10 | 2 |
| **Open surgery** | 1019 | 1128 | 755 | 401 | 204 | 113 | 62 | 31 |
| **Total** | 1794 | 2034 | 1188 | 576 | 282 | 143 | 72 | 33 |

| **Table S4. Patients demographics and surgery–related characteristics in the matched cohort for type of surgery.** | | | |
| --- | --- | --- | --- |
|  | **Laparoscopic abdominal surgery**  **(N = 254)** | **Non–laparoscopic abdominal surgery**  **(N = 344)** | **SMD** |
| **Age, years** | 54 (18) | 55 (16) | 0.070 |
| **Gender, male (%)** | 37% (94/254) | 41% (143/344) | 0.094 |
| **BMI (Kg∙m^-2^)** | 25.9 [23.4–30.1] | 26.6 [23.3–29.7] | 0.015 |
| **ASA class, % (n/N)** |  |  | 0.256 |
| 1  2  3  4  5 | 22% (55/254)  56% (143/254)  22% (56/254)  0% (0/254)  0% (0/254) | 20% (70/344)  53% (180/344)  24% (84/344)  3% (9/344)  0% (1/344) |  |
| **ARISCAT class, % (n/N)** |  |  | 0.099 |
| < 26  26–44  > 44 | 55% (141/254)  36% (91/254)  9% (22/254) | 51% (174/344)  40% (137/344)  9% (33/344) |  |
| **Current smoker, %** | 81% (205/254) | 78% (269/344) | 0.062 |
| **Chronic comorbidity, % (n/N)** |  |  |  |
| Metastatic cancer  Chronic kidney failure  COPD  Heart failure  OSAS  Neuromuscular disease^a^ | 2% (22/254)  1% (13/254)  7.% (83/254)  6% (53/254)  3% (27/254)  1% (6/254) | 10% (116/344)  6% (68/344)  6% (55/344)  8% (90/344)  1% (15/344)  1% (11/344) | 0.141  0.042  0.012  0.050  0.022  0.060 |
| **Functional Status, % (n/N)** |  |  | 0.066 |
| Independent  Partially dependent  Totally dependent | 94% (239/254)  5% (13/254)  1% (2/254) | 93% (323/344)  5% (16/344)  2% (5/344) |  |
| **Preop transfusion, % (n/N)** | 1% (3/254) | 1% (3/344) | 0.031 |
| **Surgical procedure**^b^, **% (n/N)** |  |  |  |
| Lower GI  Upper GI, HBP  Urological  Gynecological  Endocrine surgery  Neurosurgery  Other procedure | 76% (194/254)  28% (72/254)  11% (29/254)  31% (81/254)  0% (0/254)  1% (1/254)  4% (12/254) | 72% (264/344)  27% (95/344)  11% (39/344)  28% (97/344)  0% (1/344)  1% (2/344)  4% (15/344) | 0.111  0.016  0.003  0.081  0.076  0.024  0.017 |
| **Urgency of Surgery**^c^, **% (n/N)** |  |  | 0.017 |
| Elective  Urgent  Emergency | 83% (212/254)  11% (27/254)  6% (15/254) | 83% (288/344)  11% (37/344)  6% (19/344) |  |
| **Duration of anesthesia^e^ , min** | 109 [78–171] | 120 [85–180] | 0.111 |
| **Time of surgery**, **% (n/N)** |  |  | 0.001 |
| Daytime^f^  Night–time | 95% (242/254)  5% (12/254) | 95% (328/344)  5% (16/344) |  |
| **Antibiotic prophylaxis, % (n/N)** | 82% (662/254) | 85% (292/344) | 0.091 |
| **Intraop. procedures, % (n/N)** |  |  |  |
| Epidural anesthesia | 7% (18/254) | 8% (28/344) | 0.040 |
| Neuromuscular Monitoring | 19% (49/254) | 20% (71/344) | 0.034 |
| Neuromuscular Reversal | 49% (124/254) | 49% (171/344) | 0.018 |
| TIVA | 9% (24/254) | 9% (31/344) | 0.015 |
| Transfusion | 4% (10/254) | 5% (19/344) | 0.075 |
| Total Fluids (mL∙ kg^-1^) | 22 (15) | 23 (14) | 0.094 |
| Crystalloids (mL∙ kg^-1^) | 20 (14) | 21 (14) | 0.076 |
| **Ventilation mode, % (n/N)** |  |  | 0.002 |
| Volume–controlled | 82% (208/254) | 82% (282/344) |  |
| Pressure–controlled | 18% (46/254) | 18% (62/254) |  |
| **Tidal Volume** |  |  |  |
| Per ABW (ml∙kg^-1^) | 7 (1) | 7 (1) | 0.027 |
| **Minute ventilation (L∙kg^-1^)** | 6.4 (1.1) | 6.4 (1.5) | 0.023 |
| **Respiratory system compliance** |  |  |  |
| Dynamic, ml∙cm∙H_2_O^-1^ | 27 (9) | 27 (9) | 0.072 |
| Static, ml∙cm∙H_2_O^-1^ | 43 (13) | 44 (12) | 0.041 |
| **Routine recruitment maneuvers, % (n/N)** | 7% (17/254) | 7% (25/344) | 0.023 |
| **FiO_2_, %** | 55 (13) | 55 (13) | 0.008 |
| **SpO_2_, %** | 99 (1) | 99 (1) | 0.003 |
| **EtCO_2_, kPa** | 4.5 (0.6) | 4.4 (0.6) | 0.106 |
| **Airway pressures** |  |  |  |
| **Driving pressure (cm∙H_2_O)** |  |  |  |
| Time–weighted average | 8.42 (3.55) | 8.46 (3.24) | 0.009 |
| Maximum value | 15.03 (5.03) | 14.75 (4.79) | 0.058 |
| Minimum value | 11.81 (4.20) | 11.80 (4.32) | 0.002 |
| Coefficient of variation (%) | 13.21 (12.83) | 12.47 (11.64) | 0.061 |
| **PEEP (cm∙H_2_O)** |  |  |  |
| Maximum value | 3.72 (2.45) | 3.72 (2.61) | 0.001 |
| Minimum value | 2.87 (2.29) | 2.87 (2.34) | 0.001 |
| Data are presented as meas (SD) or median [25^th^–75^th^ percentile] or % (n/N).  Abbreviations: BMI, body mass index; ASA, American Society of Anesthesiologists; ; GI: gastrointestinal; HBP, Hepatobiliopancreatic; SpO_2_, peripheral oxygen saturation: CI, confidence interval; SMD, Standardized mean differences.  ^a^Neuromuscular disease affecting the respiratory system.  ^b^Tha same patient may have more than one surgical indication  ^c^Urgency of surgery is defined as *elective*: surgery that is scheduled in advance because it does not involve a medical emergency, *urgent*: surgery required within <48 hours*, emergent*: surgery performed when the patients' life or well being are threatened.  ^d^Duration of surgery is the time between skin incision and closure of the incision.  ^e^Duration of anaesthesia is the time between start of induction and tracheal extubation or discharge from operation room if the mechanical ventilation is continued.  ^f^Daytime surgery is defined as anaesthesia induction between 8:00 a.m. and 19:59 p.m. | | | |

| Table S5. Intraoperative and postoperative outcomes in matched cohort for type of surgery. | | | |
| --- | --- | --- | --- |
|  | **Closed surgery**  **(N = 254)** | **Open surgery**  **(N = 341)** | ***P–* value** |
| Severe PPC (composite), % (n/N) | 4% (10/254) | 7% (22/344) | 0.246 |
| Intraoperative complications |  |  |  |
| Desaturation | 1% (3/254) | 4% (16/344) | 0.030 |
| Unplanned rescue maneuvers | 3% (8/254) | 4% (16/344) | 0.986 |
| Airway pressure reduction needed | 7% (18/254) | 1% (4/344) | < 0.001 |
| Expiratory flow limitation | 0.3% (4/254) | 1% (1/344) | 0.215 |
| Hypotension | 20% (53/254) | 25% (380/344) | 0.192 |
| Use of vasopressors | 17% (43/254) | 20% (68/344) | 0.408 |
| New arrhythmia onset | 0% (0/254) | 1% (3/344) | 0.361 |
| Individual PPCs |  |  |  |
| Unplanned need for supplementary O_2_ | 13% (33/254) | 15% (52/344) | 0.509 |
| Acute respiratory failure | 3% (9/254) | 3% (12/344) | 1.000 |
| Need for mechanical ventilation | 3% (8/254) | 3% (11/344) | 1.000 |
| Acute respiratory distress syndrome | 0% (0/254) | 0.6% (2/344) | 0.612 |
| Pneumonia | 0% (0/254) | 1% (5/344) | 0.138 |
| Pneumothorax | 0% (0/254) | 0.3% 1/344) | 1.000 |
| Data are presented as median [25^th^–75^th^ percentile] or % (n/N). PPC, postoperative pulmonary complications. | | | |

| **Table S6. Mixed multivariable logistic regression in matched cohort for postoperative pulmonary complications** | | |
| --- | --- | --- |
| Variables | **OR** [**L– U 95%CI]** | ***P*** |
| **Laparoscopic surgery Yes** | **0.69 [0.39 to 1.21]** | **0.238** |
| **Time–weighted driving pressure** | 0.44 [0.19 to–0.97] | 0.043 |
| **Maximum driving pressure** | 5.714 [1.57 to 20.740] | 0.008 |
| **Driving pressure coefficient of variation** | 0.66 [0.344 to 1.27] | 0.210 |
| **Minimum driving pressure** | 0.46 [0.168 to 1.26] | 0.130 |
| **Maximum PEEP** | 1.03 [0.66 to 1.60] | 0.890 |
| **Minimum PEEP** | 1.47 [0.95 to 2.28] | 0.081 |
| **Age** | 1.33 [0.95 to 1.87] | 0.100 |
| **Gender** | 0.86 [0.48 to 1.56] | 0.621 |
| **Asa (Reference: ASA 1)** |  |  |
| Asa 2 | 1.37 [0.531 to 3.530] | 0..516 |
| Asa 3 | 2.09 [0.71 to 6.07] | 0.178 |
| Asa ≥ 4 | 4.721 [0.63 to 34.946] | 0.129 |
| **Chronic Bronchitis (Yes)** | 3.40 [1.28 to 8.980] | 0.014 |
| **Cancer (Yes)** | 2.33 [1.04 to 5.25] | 0.040 |
| **Duration of anesthesia** | 1.96 [1.30 to 2.97] | 0.001 |
| **ARISCAT score (Reference: Low < 26 )** |  |  |
| ARISCAT Intermediate (26 – 44) | 0.85 [0.44 to 1.63] | 0.621 |
| ARISCAT high (>45) | 1.26 [0.48 to 3.30] | 0.640 |
| **Urgency of Surgery (Reference: Elective)** |  |  |
| Urgent | 0.55 [0.18 to 1.68] | 0.291 |
| Emergency | 3.47 [0.99 to 12.070] | 0.051 |
| **Epidural anesthesia** | 1.13 [0.43 to 2.97] | 0.797 |
| **NMB monitoring Yes** | 1.52 [0.73 to 3.15] | 0.260 |
| **TV Per body weight** | 0.80 [0.59 to 1.10] | 0.169 |
| **Static Compliance** | 0.94 [0.56 to 1.58] | 0.816 |
| Sd centers. (Intercept) | 0.97 | |
| Abbreviations: OR, Odds Ratio; L, Lower 95% Confidence Interval Boundary; U 95% Confidence Interval Boundary | | |

**Figure S1.** Time weighted average and coefficient of variation calculation

**Figure S2.** Summary plot of covariate balance for time‑weighted ΔP before (red line) and after (blue line) conditioning for open surgery.

**Figure S3.** Summary plot of covariate balance before (red line) and after (blue line) conditioning for closed surgery. **A**: time–weighted; **B**: Highest value; **C:** Lowest Value; **D**: Coefficient of variation.

**Figure S4.** Residuals plot for postoperative pulmonary complications (PPCs) and intraoperative adverse events (AEs). A: PPCs in Open surgery; B: PPCs in closed surgery; C; AEs in open surgery; D: AEs in closed surgery.

**REFERENCES**

1. Dunkler D, Plischke M, Leffondré K, Heinze G. Augmented backward elimination: A pragmatic and purposeful way to develop statistical models. *PLoS One*. 2014; **9**:1–19.
2. Ho D, Imai K, King G, Sturat EA. MatchIt: Nonparametric Preprocessing for Parametric Causal Inference. *J Stat Softw*. 2011. **42**:pii: 8.
